# Fine-scale heterogeneity and local amplification of West Nile virus in urban environments in Berlin

**DOI:** 10.1038/s41467-026-73251-5

**Published:** 2026-06-12

**Authors:** Corinna Patzina-Mehling, Anne Kopp, Yea-Seul Lee, Maximillian X. L. Birkl, Sophia Ebers, Katrin Voigt, Selina L. Graff, Uli Beisel, Conny Landgraf, Florian Ganz, Aimara Planillo, Stephanie Kramer-Schadt, Sandra Junglen

**Affiliations:** 1 Institute of Virology, Charité – Universitätsmedizin Berlin, corporate member of Freie Universität Berlin and Humboldt-Universität zu Berlin, Berlin, Germany; 2 German Centre for Infection Research (DZIF), Partner Site Charité, Berlin, Germany; 3 Institute of Geographical Sciences, Freie Universität Berlin, Berlin, Germany; 4 Department of Ecological Dynamics, Leibniz Institute for Zoo and Wildlife Research, Berlin, Germany; 5 Department of Conservation Biology and Global Change, Doñana Biological Station (EBD-CSIC), Seville, Spain; 6 Institute of Ecology, Technische Universität Berlin, Berlin, Germany

**Keywords:** West nile virus, Urban ecology, Epidemiology

## Abstract

Climate change can intensify mosquito-borne disease risks through rising temperatures and more frequent extreme weather events. To mitigate effects of climate change, cities are adopting nature-based solutions, such as urban greening and rainwater management, yet their implications for vector-borne diseases and host community composition remain poorly understood. West Nile virus (WNV), an emerging mosquito-borne human pathogen in Europe, is primarily transmitted between birds and mosquitoes. Using mosquito sampling at five sites within a one-square-kilometre area in Berlin, Germany, we examined how urban land cover, including climate-resilient infrastructure, influences local WNV amplification over two mosquito seasons in 2023 and 2024. We found seasonal WNV infection rates of up to 4.8% in mosquitoes and identified fine-scale heterogeneity in infection risk. Residential areas and cemeteries exhibited the highest minimum infection rates per month (up to 15 and 21, respectively), whereas natural conversation and sponge city sites showed significantly lower rates (up to 4 and 13, respectively). These patterns were not explained by mosquito abundance or species composition but by habitat characteristics and avian host community structure. Our findings reveal that urban land cover shapes WNV infection risk and suggest that incorporating biodiversity restoration into nature-based solutions may serve as strategy for sustainable climate-resilient urban planning.

## Abstract (web summary)

Mosquito-borne infections such as West Nile Virus are emerging in Europe. Here, the authors investigate how West Nile Virus infection rates in mosquitoes vary in different urban environments in Berlin.

## Introduction

Cities worldwide face growing challenges due to high population density, low biodiversity and increasing vulnerability to climate change, creating an urgent need for sustainable adaption strategies^1–3^. In response, many urban areas are adopting nature-based solutions, such as urban greening and rainwater management, in line with the United Nations Environment Programme^1^. These approaches enhance climate resilience and biodiversity while improving human well-being. Europe has launched major policy frameworks, including the Green Deal^2^ and the Biodiversity Strategy 2030^3^, to address environmental and climate challenges. While such initiatives offer multiple benefits, their implications for vector-borne diseases remain poorly understood. Expanding green and blue spaces can unintentionally create suitable habitats for mosquitoes and vertebrate hosts, potentially increasing spillover infection risk to humans. Yet, empirical evidence linking nature-based solutions with vector-borne disease risks is scarce, underscoring the need for systematic evaluation^4,5^.

Mosquito-borne viruses such as dengue, chikungunya and West Nile virus (WNV) are increasingly reported in urban and peri-urban environments, representing a growing public health burden under changing climatic conditions^6–8^. While knowledge of the fine-scale spatial distribution of *Aedes*-borne infections in urban areas is increasing, and areas with persistent geographical transmission hotspots have been identified^9–13^, understanding of *Culex*-borne infections is lagging behind. Among these, WNV is of particular relevance in Europe due to its ongoing emergence and projected expansion under climate change^7,14–17^. Mosquitoes acquire WNV from infected birds and transmit it to humans, who may develop disease but do not contribute to viral transmission. Symptoms of disease include West Nile fever (WNF), or, in rare cases, a neuroinvasive disease (WNND) with a case-fatality rate of approximately 10%^18,19^.

The ecology of WNV is complex and shaped by environmental and climatic factors. However, key aspects of its amplification, particularly in urbanised habitats and in the context of climate adaptation strategies, remain insufficiently understood. The potential effects of climate-resilient infrastructure and nature-based solutions on mosquitoes and bird community composition, and consequently on local WNV amplification, are largely unexplored.

WNV transmission depends on the abundance and species composition of competent vectors and avian hosts. In Europe, the main vectors are *Culex (Cx.) pipiens* complex mosquitoes, which thrive in urban environments, feed on birds and humans and breed in nearly every type of water source^20^. While peridomestic birds, such as the American robin (*Turdus migratorius*), the house finch (*Haemorhous mexicanus*) and the house sparrow (*Passer domesticus*), have been identified as the key amplification hosts in North America^21–23^, data on the amplification competence of European bird species remain limited^24–26^. Diverse bird communities containing non-competent hosts can reduce transmission through a “dilution effect”, whereby vectors feed less frequently on amplifying species^27^. However, evidence for this effect for WNV is mixed^28–30^. Conversely, high mosquito and avian host densities are often positively correlated with mosquito infection rates and clusters of human cases^31^, with mosquito infection rates serving as strong predictors of human cases^32,33^.

Abiotic factors, including temperature, precipitation and land-cover, also play key roles in shaping mosquito populations. Higher temperatures accelerate mosquito development, biting rates and WNV replication in vectors^34–36^. Abnormally high temperatures have been associated with increased WNV incidence in both North America and Europe^37–40^. Precipitation provides breeding habitats for mosquitoes and correlates with higher vector abundance and human WNV cases^41,42^. Extreme weather events can have opposing effects, as heavy rainfall may wash out breeding sites^43,44^, whereas drought can increase contact between birds and mosquitoes at remaining natural water bodies as well as at anthropogenic water sources, such as irrigation systems and water storage sites^45–47^. Land cover determines habitat suitability and food availability, influencing both mosquito and bird community composition^48–51^. WNV abundance tends to be highest in urban and agricultural landscapes^15,45,52,53^, yet the fine-scale ecology of WNV in European urban settings remains poorly resolved, as most studies have contrasted broader land cover categories, e.g. urban versus rural^54^.

In Germany, WNV has become endemic in the Berlin metropolitan area, with recurring detections in mosquitoes, birds and humans^55–58^. Berlin provides a valuable case study, as it is actively implementing climate adaption measures, such as sponge city projects, an urban planning approach that enhances rainwater retention and infiltration through decentralised rainwater management, including the installation of permeable surfaces and natural drainage systems (Berliner Regenwasseragentur, https://regenwasseragentur.berlin/), the Green Roof programme (Berliner GründachPLUS Programm, 2026-01), and the Berlin Tree Planting Law, which aims to plant over one million trees by 2040 (Berliner Klimaanpassungsgesetz, 2025-11-07).

In this study, we investigate at a fine spatial scale how urban land cover and nature-based solutions affect mosquitoes and bird species assemblages and local WNV amplification within an area of Berlin where WNV has recently established endemicity^55^. We further link these outcomes to microhabitat characteristics to identify urban features that may enhance or mitigate WNV infection risk.

## Results

### Urban microhabitats show distinct patterns of mosquito abundance and WNV infection

**Fig. 1:**
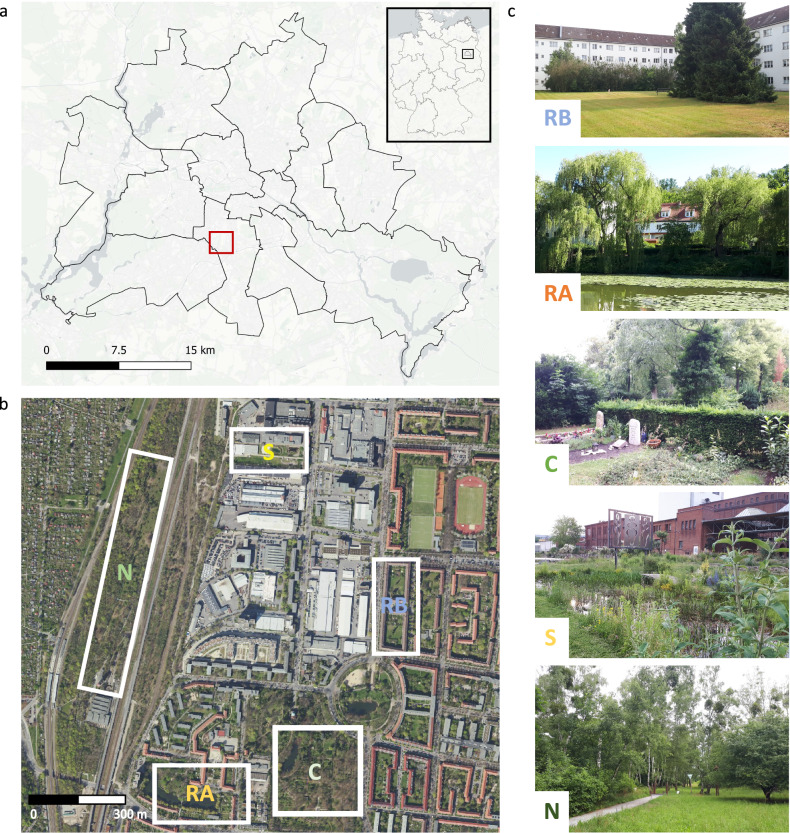
Sampling sites of this study. **a** Map of Berlin, Germany, with the sampling area in the district of Tempelhof-Schöneberg shown in a red box (created with QGIS 3.4.12). **b** Aerial picture of sampling locations (from 2023, adapted from Berlin geoportal; https://gdi.berlin.de/viewer/main/). C cemetery, N nature conservation site, RA residential area, RB residential block, S sponge city site. **c** Pictures taken at the sampling sites.

**Fig. 2:**
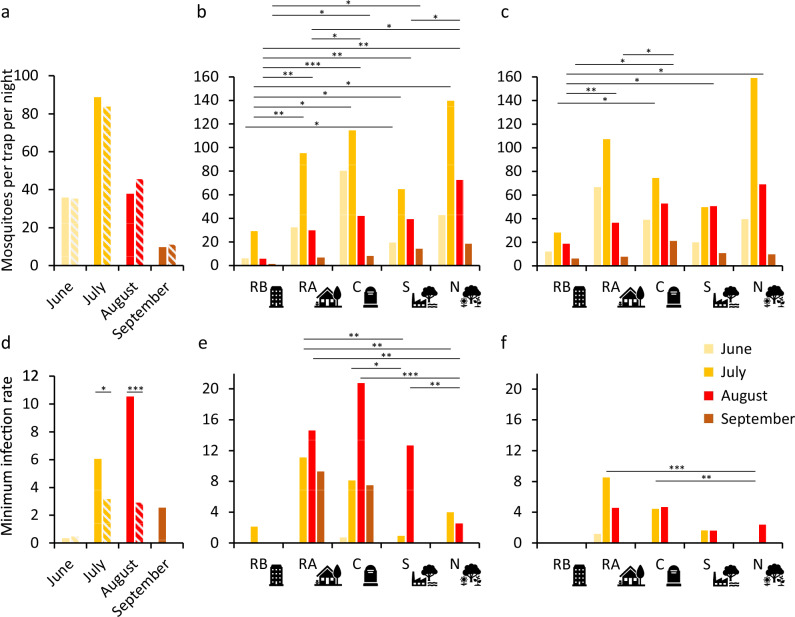
Mosquito abundance and WNV minimum infection rates (MIRs). **a** Number of collected mosquitoes were normalised to trap performance per catch night and are shown according to sampling month and year (filled bars, 2023; striped bars, 2024). **b**, **c** Normalised number of mosquitoes per site and month in 2023 (**b**) and 2024 (**c**) (two-sided unpaired Welch’s *t*-test; * *p* < 0.05; ** *p* < 0.01; *** *p* < 0.001; in detail: RB vs. S June 2023 *p* = 0.0326, RB vs. RA July 2023 *p* = 0.0075, RB vs. C July 2023 *p* = 0.0141, RB vs. S July 2023 *p* = 0.0195, RB vs. N July 2023 *p* = 0.0410, RB vs. RA August 2023 *p* = 0.0034, RB vs. C August 2023 *p* < 0.0001, RB vs. S August 2023 *p* = 0.0020, RB vs. N August 2023 *p* = 0.0073, RB vs. C September 2023 *p* = 0.0367, RB vs. S September 2023 *p* = 0.0159, RA vs. C August 2023 *p* = 0.0335, RA vs. N August 2023 *p* = 0.0167, S vs. N August 2023 *p* = 0.0403, RB vs. C July 2024 *p* = 0.0464, RB vs. RA August 2024 *p* = 0.0045, RB vs. S August 2024 *p* = 0.0453, RB vs. N August 2024 *p* = 0.0368, RB vs. C September 2024 *p* = 0.0295, RA vs. C September 2024 *p* = 0.0290). **d** MIRs were calculated as WNV-*p*ositive mosquito pools per total numbers of mosquitoes per month per 1000 individuals and shown according to sampling month and year (filled bars, 2023; striped bars, 2024). **e**, **f** MIRs per site per month in 2023 (**e**) and 2024 (**f**). To analyse statistical significance, a two-way Chi-Squared test was performed (** *p* < 0.01; *** *p* < 0.001; in detail: July 2023 vs. 2024 *p* = 0.0248, August 2023 vs. 2024 *p* = 0.0005, RA vs. S July 2023 *p* = 0.0025, RA vs. N July 2023 *p* = 0.0097, RA vs. N August 2023 *p* = 0.0044, C vs. S July 2023 *p* = 0.0174, C vs. N August 2023 *p* = 0.0001, S vs. N August 2023 *p* = 0.0089, RA vs. N July 2024 *p* = 0.0001, C vs. N July 2024 *p* = 0.0034). Source data are provided in the Source Data file. Months are colour-coded: June, yellow; July, orange; August, red; September, brown. Icons were adapted from Microsoft PowerPoint.

A total of 13,627 and 10,463 mosquitoes were collected across five urban microhabitats in 2023 and 2024, respectively, using a standardised trapping system (Fig. 1). In both years, mosquito abundance peaked in July and was lowest in September (Fig. 2a), and a similar mosquito abundance pattern for the five microhabitats was found. The highest abundance was observed at a nature conservation area (N), followed by a park-like cemetery (C) and a residential area (RA), while the lowest was recorded at a residential backyard (RB) (Fig. 2b, c, Table 1). The sponge city site (S) did not exhibit elevated mosquito abundance in both years and had lower counts than RA, C and N, despite having permanent open water similar to RA and C.

**Table 1:** Total number of mosquitoes per species per site per month

		June					July					August					September				
		RB	RA	C	S	N	RB	RA	C	S	N	RB	RA	C	S	N	RB	RA	C	S	N
*Culex pipiens* s.s.	2023	78	460	1008	268	446	296	1387	1466	890	2063	54	279	560	393	654	13	94	58	171	215
	2024	98	756	372	171	356	300	1178	717	549	1790	179	374	445	541	652	69	76	163	117	96
*Culex mimeticus*	2023	0	0	0	0	0	0	0	0	0	0	0	0	0	0	0	0	0	0	0	0
	2024	0	0	0	0	4	0	0	0	0	0	0	0	0	0	0	0	0	0	0	0
*Culex modestus*	2023	0	1	0	2	0	0	4	6	6	0	0	4	0	0	0	0	1	1	0	0
	2024	0	1	1	3	0	3	26	24	4	7	1	1	33	13	7	0	1	0	0	0
*Culex* sp.	2023	11	27	257	39	25	147	83	301	102	60	13	145	46	188	382	4	9	14	53	42
	2024	0	0	0	24	0	28	60	52	37	43	2	38	15	31	4	0	8	11	8	5
*Culiseta annulata*	2023	0	1	0	0	1	2	6	2	3	2	0	3	0	2	0	0	0	1	0	1
	2024	0	0	1	0	0	0	5	1	3	1	0	0	0	0	3	0	3	2	0	1
*Culiseta morsitans*	2023	0	0	0	0	0	0	0	1	0	0	0	0	1	0	0	0	0	1	0	0
	2024	0	0	0	0	1	0	0	0	0	0	0	0	1	0	0	0	1	1	0	0
*Culiseta sp*.	2023	0	0	0	0	0	0	0	0	0	0	0	0	0	0	0	0	0	0	0	0
	2024	1	0	1	0	0	0	0	0	0	0	0	0	0	0	0	0	0	0	0	0
*Coquillettidia richardii*	2023	1	20	3	1	12	0	19	2	1	11	0	4	2	0	1	0	0	0	0	0
	2024	0	16	5	0	7	0	9	1	0	6	0	1	0	0	6	0	0	0	0	0
*Aedes vexans*	2023	0	1	0	0	1	5	16	16	26	50	6	8	4	10	10	2	0	0	3	7
	2024	32	21	7	21	19	1	1	3	3	6	41	15	21	13	60	0	1	0	0	1
*Aedes cataphylla*	2023	0	0	0	0	0	0	0	0	0	0	0	0	0	0	0	0	0	0	0	0
	2024	0	0	0	1	0	0	0	0	0	0	0	0	0	0	0	0	0	0	0	0
*Aedes cinereus/geminus*	2023	0	1	1	0	0	7	2	9	4	11	7	4	15	7	21	0	0	1	0	0
	2024	0	0	0	0	1	1	0	2	0	6	2	1	5	0	3	0	0	0	0	0
*Aedes rossicus*	2023	0	0	0	0	0	0	1	0	1	0	0	0	0	0	0	0	0	0	0	0
	2024	0	0	0	0	0	0	0	0	0	0	0	1	0	1	0	0	0	0	0	0
*Aedes caspius*	2023	0	0	0	0	0	0	1	0	0	0	3	1	0	0	2	0	0	0	0	0
	2024	1	1	0	5	0	1	0	0	0	0	1	3	0	2	1	0	0	0	0	0
*Ochlerotatus geniculatus*	2023	1	2	3	0	8	0	4	11	1	21	1	1	13	1	9	0	1	4	0	6
	2024	0	1	4	0	3	1	1	9	0	9	0	1	2	0	8	0	0	3	0	0
*Aedes annulipes/cantans*	2023	5	3	4	2	9	0	0	2	1	3	0	0	1	0	0	0	0	0	0	0
	2024	1	1	7	2	8	0	0	0	0	1	0	0	0	0	0	0	0	0	0	0
*Ochlerotatus sticticus*	2023	0	0	0	0	0	0	0	1	0	0	1	1	0	0	1	0	0	0	0	0
	2024	0	0	0	0	0	0	0	0	0	0	0	1	1	0	0	0	0	0	0	0
*Aedes*/*Ochlerotatus* sp.	2023	0	0	0	0	3	7	0	4	0	0	0	3	0	3	6	0	0	0	0	0
	2024	11	3	9	11	11	1	0	1	0	1	0	0	3	3	3	0	0	1	0	0
*Anopheles plumbeus*	2023	1	0	9	0	11	0	0	10	1	9	1	5	32	0	24	0	2	51	0	23
	2024	0	1	20	1	26	2	6	79	3	37	0	1	109	1	79	0	1	68	4	13
*Anopheles claviger*	2023	0	0	0	0	0	0	0	0	0	0	0	0	0	0	0	0	0	0	0	0
	2024	0	0	0	0	0	0	1	0	0	0	0	0	0	1	2	0	0	1	0	0
*Anopheles* sp.	2023	0	0	0	0	0	0	0	4	0	1	0	0	0	0	2	0	0	0	0	0
	2024	0	0	0	0	0	0	0	3	0	0	0	0	0	0	0	0	0	3	1	0
unidentified	2023	0	0	0	0	0	0	0	0	0	0	3	20	0	260	49	0	0	2	0	0
	2024	0	0	1	0	0	0	0	0	0	1	0	0	0	0	0	0	0	0	0	0
# total	2023	97	516	1285	312	516	464	1523	1835	1036	2231	89	478	674	630	1161	19	107	133	227	294
	2024	144	801	428	239	436	338	1287	892	599	1908	226	437	635	606	828	69	91	253	130	116
Species total	2023	*5*	*8*	*6*	*4*	*7*	*4*	*9*	*11*	*10*	*8*	*7*	*10*	*8*	*5*	*8*	*2*	*4*	*7*	*2*	*5*
	2024	*5*	*8*	*8*	*7*	*9*	*7*	*8*	*8*	*5*	*9*	*5*	*10*	*8*	*7*	*10*	*1*	*6*	*6*	*2*	*4*

The number of individual mosquitoes per species for each site and month in 2023 and 2024 is shown. *s.s.* subspecies, *sp.* species.

In 2023, WNV infection in mosquitoes was high, with 4.8 % of mosquito pools testing positive (78/1,625). For all sites except N, minimum infection rates (MIRs) peaked in August, with the highest MIRs observed at C (20.77) and RA (14.60) (Table 2, Fig. 2d, e), reaching levels comparable to those reported from outbreak areas in southern Europe^59–61^. In contrast, infection rates at N remained low, particularly in August (2.58). In July, MIRs were lowest at S (0.97) and N (4.03) and significantly higher at RA (11.16) and C (8.17). In September, WNV was detected only at RA and C, with comparatively high MIRs, whereas RB showed the lowest infection risk, with only a single WNV detection in July.

**Table 2:** WNV-positive mosquito pools identified per site and month in the respective sampling year

		June			July			August			September			Total
Site	Sampling year	#	% pos.	MIR	#	% pos.	MIR	#	% pos.	MIR	#	% pos.	MIR	#
RB	2023	0	0	0	1	1.96	2.16	0	0	0	0	0	0	1
	2024	0	0	0	0	0	0	0	0	0	0	0	0	0
RA	2023	0	0	0	17	10.69	11.16	7	9.86	14.64	1	4.55	9.35	25
	2024	1	1.10	1.25	11	7.43	8.55	2	3.70	4.58	0	0	0	14
C	2023	1	0.67	0.78	15	7.69	8.17	14	15.91	20.77	1	3.57	7.52	31
	2024	0	0	0	4	3.77	4.48	3	4.11	4.72	0	0	0	7
S	2023	0	0	0	1	0.90	0.97	8	10.13	12.70	0	0	0	9
	2024	0	0	0	1	1.45	1.67	1	1.39	1.65	0	0	0	2
N	2023	0	0	0	9	3.93	4.03	3	2.27	2.58	0	0	0	12
	2024	0	0	0	0	0	0	2	2.13	2.42	0	0	0	2
Total	2023	1	0.28	0.37	43	5.77	6.07	32	8.14	10.55	2	1.50	2.56	78
	2024	1	0.36	0.49	16	2.71	3.18	9	2.48	2.93	0	0	0	25

Total number of WNV detection (#), percentage of WNV positive pools (% pos.) and MIR per site and month in 2023 and 2024 are listed.

WNV infection rates of mosquitoes were significantly lower in 2024, with only 1.95 % of the mosquito pools (25/1280) testing positive for WNV. Again, higher MIRs were detected at RA and C compared to N and S (Fig. 2f, Table 2). WNV peaked at RA (8.55) in July and was only detected at N (2.42) in August, whereas lower but similar levels were found at C (4.48–4.72) and S (1.67–1.65) in July and August. Again, the highest MIRs were detected at RA and C in both months, while WNV was lower at S and significantly lower at N, where it was only detected in August. WNV was detected once at RA in June, with no detections at RB throughout the sampling season and none at any site in September.

### WNV amplified locally confirmed by genomic surveillance

**Fig. 3:**
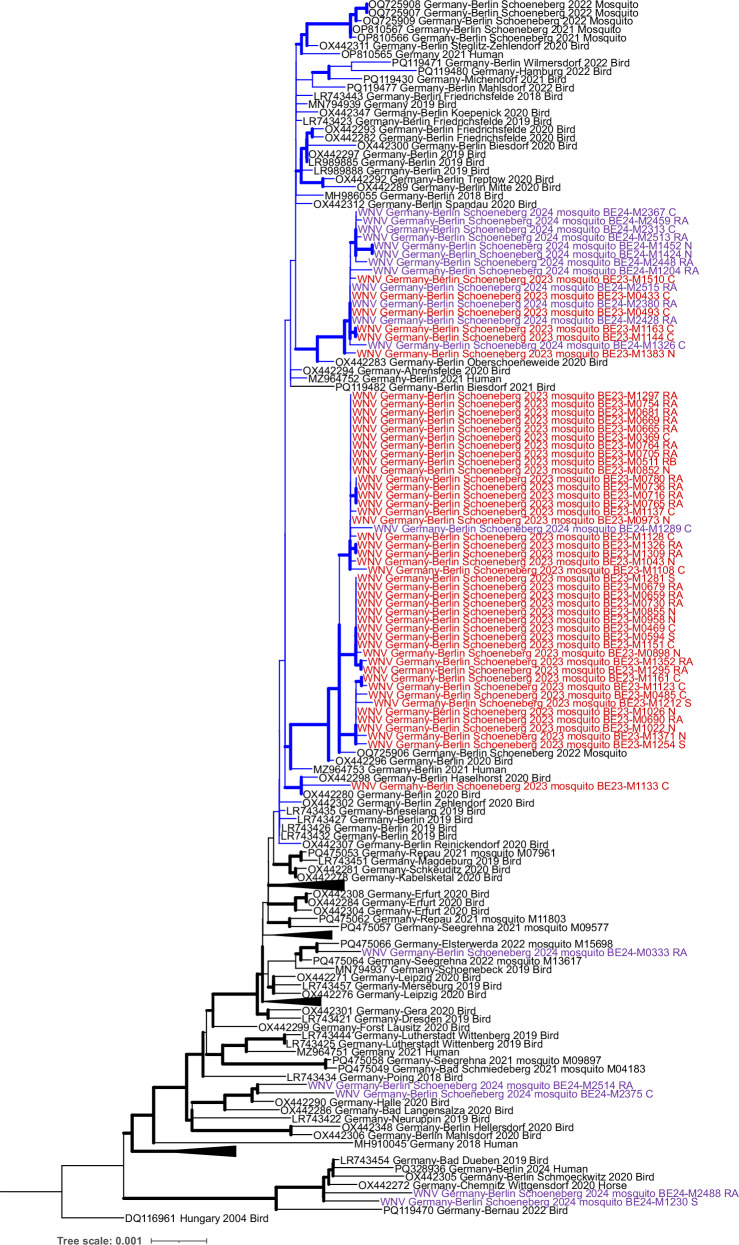
Phylogenetic analysis of WNV sequences from this study. The phylogenetic relationship of 67 coding-complete WNV sequences from the five study sites was inferred using a maximum likelihood (PHYML) tree based on a MAFFT alignment using the TN93 substitution model and 1000 bootstrap replicates (thick lines highlight clades with bootstrap values above 75). The Berlin clade is marked in blue. Sequences from this study are marked in red (2023) and violet (2024). Triangles indicate collapsed subtrees. Source data are provided in the Source Data file.

We performed NGS on all WNV-positive samples, obtaining complete coding sequences for 49 WNV strains from 2023 and 18 from 2024. Samples with fewer than 600,000 genome copies per mL (ct values  >29) did not yield complete coding sequences. For these samples, nested PCRs and Sanger sequencing were performed, yielding partial sequences from 23 strains from 2023 and 4 from 2024, covering 0.6–5 kb of the WNV coding sequence. Genome analysis revealed that all detected WNV sequences carried the unique Berlin-specific nucleotide variants, with the exception of five sequences from 2024. Phylogenetic analysis of complete coding sequences placed these five sequences within the broader phylogenetic diversity of WNV sequences detected across Germany, whereas all sequences containing the Berlin-specific nucleotide variants grouped within the Berlin clade, supporting local viral amplification (Fig. 3). These sequences further resolved into two major subclades, while one sequence grouped with bird-derived WNV sequences from 2020. The largest subclade comprised 43 sequences from this study, one sequence from a bird, and two WNV sequences derived from a human case and a mosquito, both from an allotment garden in the same neighbourhood, detected in 2021 and 2022^55^. These sequences shared nine unique single-nucleotide variations (SNVs). The second subclade included six WNV sequences from 2023 and twelve from 2024 that shared four unique SNVs with a WNV sequence from a bird found in Berlin, Oberschöneweide, in 2020. Collectively, these findings indicate ongoing local WNV persistence and microhabitat-level amplification.

### WNV infection rates were independent of mosquito abundance and species composition

**Fig. 4:**
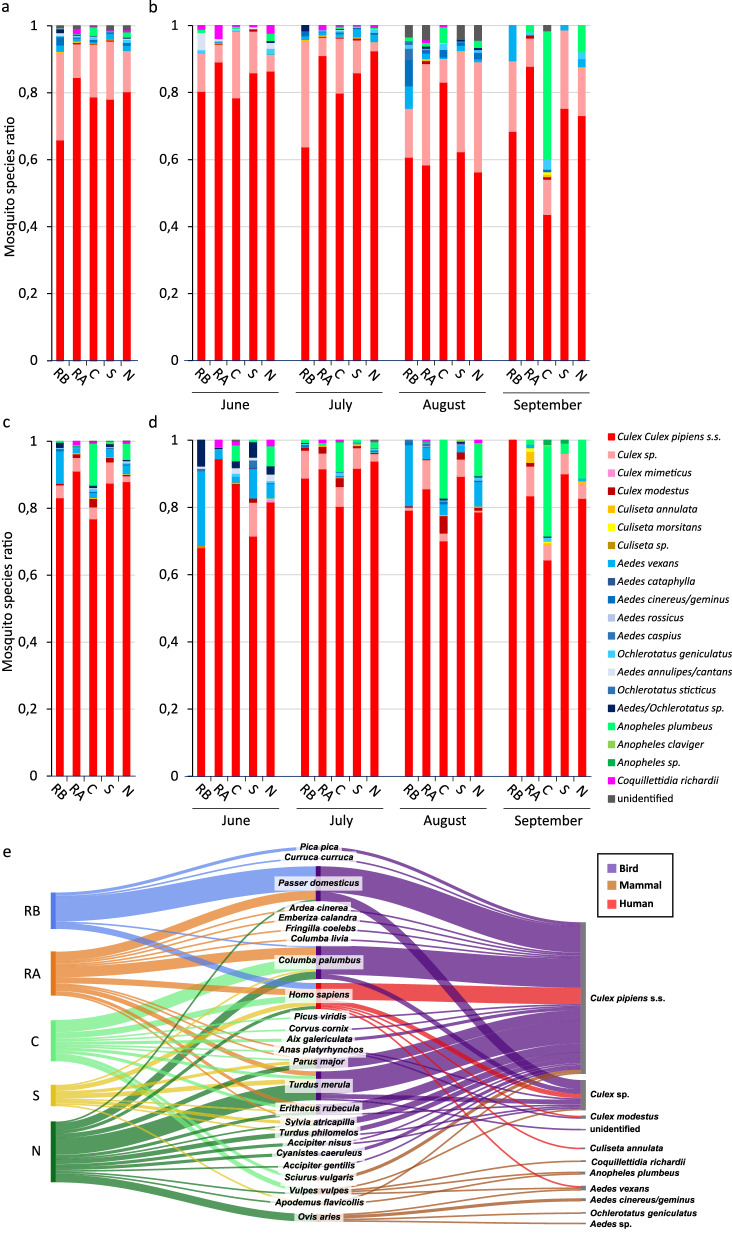
Mosquito species community composition and host sources of blood-fed mosquitoes per sampling site. **a**, **c** Cumulative total rates of mosquito species per site in 2023 (**a**) and 2024 (**c**). **b**, **d** Mosquito species assemblages per site per month in 2023 (**b**) and 2024 (**d**). Mosquito species are colour-coded as indicated in the legend. **e** Bloodmeal analysis of 134 mosquito pools containing at least one blood-fed specimen. Host sources are marked as birds (purple), humans (red) and other mammals (brown). Source data are provided in the Source Data file. Abbreviations are: s.s. subspecies, sp. species.

In total, 16 species or species complexes were identified, with *Culex pipiens* complex as the primary WNV vector representing 82.4 % of all specimens and dominating at all sites in both years (Fig. 4a, c, Table 1). Mosquitoes of the genus *Culex* were the most abundant at all sites, comprising 83.0–96.3 % of specimens per site. Other species did not exceed 12.5% per site. Common non-*Culex* species included *Aedes vexans* (0.5–9.5%), *Aedes cinereus* (0.04–2.1%), *Ochlerotatus geniculatus* (0–1.1%), *Coquillettidia richardii* (0–1.6%) and *Anopheles plumbeus* (0–12.5%).

**Fig. 5:**
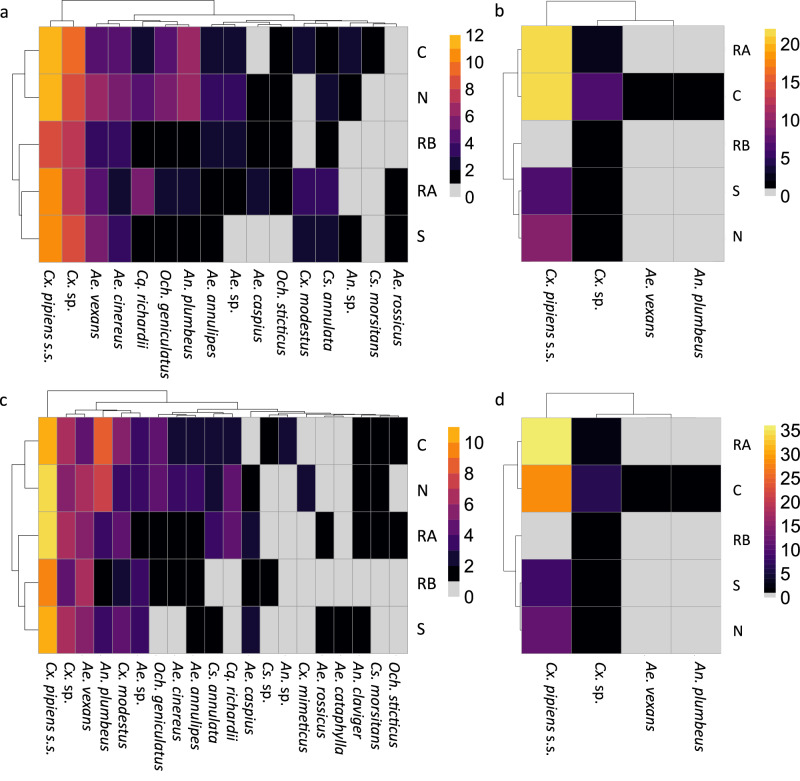
Cluster analysis of the mosquito community. **a** Hierarchical cluster analysis heatmap for individual mosquito species from 2023 according to their abundance. Colours indicate abundances [log2-scale]. Parentheses indicate clusters of similar mosquito abundances. **b** Cluster analysis heatmap for mosquito species according to the number of WNV detections in 2023. Colours indicate total abundances. Parentheses indicate clusters of similar infection strength. **c** Cluster analysis heatmap for individual mosquito species from 2024 according to their abundance. **d** Cluster analysis heatmap for mosquito species according to the number of WNV detections in 2023 and 2024 combined. Source data are provided in the Source Data file. *Cx*. *Culex*, *Cs*. *Culiseta*, *Cq*. *Coquillettidia*, *Ae*. *Aedes*, *Och*. *Ochlerotatus*, *An*. *Anopheles*, s.s. subspecies, sp. species.

No significant differences were observed in species richness, evenness or Shannon diversity index across sites (Supplementary Table 1), and rarefaction plots indicated similar mosquito species diversity (Supplementary Fig. 1). Monthly comparisons revealed minor site-specific differences in mosquito species composition. For example, *Coquillettidia richardii* occurred almost exclusively at RA, C and N in June and July. In contrast, *Anopheles plumbeus* was more abundant at C (38.3% and 26.9%) and N (7.8% and 11.2%) in September 2023 and 2024, respectively. RB showed higher proportions of *Aedes* sp. mosquitoes in June (6.2% and 31.3%) and August (20.2% and 19.5%) of 2023 and 2024, respectively (Fig. 4b, d, Table 1). Cluster analysis of mosquito species assemblages across the five sites indicated similarity between the two more natural sites N and C and the two more urban sites S and RA, with the urban backyard RB linked to S and RA in both years (Fig. 5a, c). Overall, WNV infection patterns were not explained by mosquito abundance or species composition, indicating other ecological drivers.

### *Culex* mosquitoes predominantly feed on birds, with occasional human hosts

A small proportion of collected mosquitoes were blood-fed (*n* = 199) and analysed for vertebrate host DNA, yielding 134 host sequences (Source Data). The majority (*n* = 122) originated from *Culex* mosquitoes, which fed predominantly on birds (*n* = 103), but also on humans (*n* = 15), squirrels (*n* = 2), a mouse (*n* = 1) and a fox (*n* = 1) (Fig. 4e). Humans were identified as feeding source for *Culex* at RA and C, the two sites with the highest WNV infection rates in mosquitoes, as well as at S, N and RB. In contrast, *Aedes*, *Anopheles* and *Coquillettidia* species fed on sheep, foxes, a mouse, and also on humans. House sparrows (*Passer domesticus*) were detected as the main bloodmeal sources at RB, whereas more diverse feeding patterns were observed at larger, greener sites such as RA, C and N. Three mosquito pools that contained DNA from *Passer domesticus* (*Culex* sp., RA, *n* = 2) or *Parus major* (*Culex pipiens*, C, *n* = 1) were tested positive for WNV. These bird species were also detected with the highest abundances during a field survey conducted at these sites in 2025 (Supplementary Figs. 2–4, Supplementary Methods). These results highlight site-specific differences in avian host feeding patterns of *Culex* sp., with birds as the primary bloodmeal source and occasional human exposure.

### Urban microhabitat shapes avian communities and mosquito infections

**Fig. 6:**
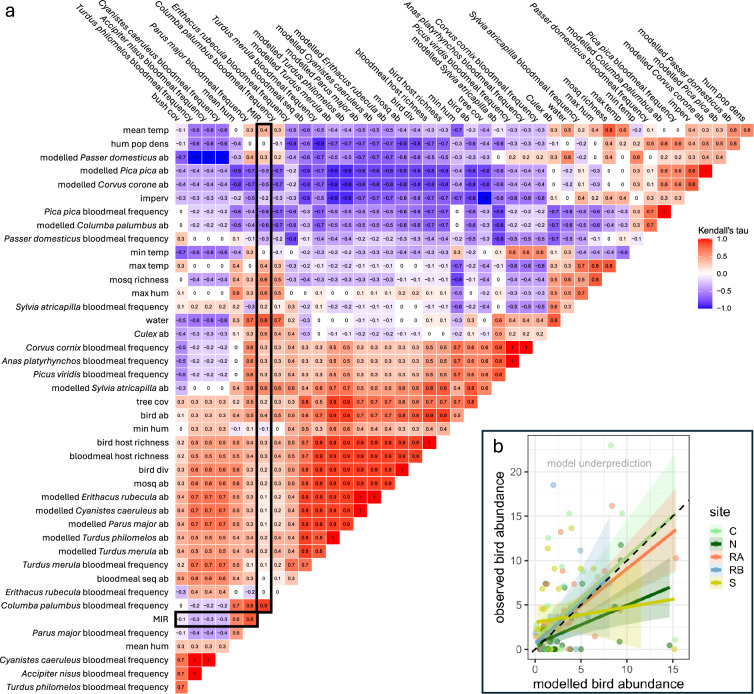
Correlation analysis of variables associated with mosquito MIR in 2023. **a** Variables from field sampling in 2023, from a spatial model on local bird abundances, as well as from the environment, were used for an extensive Kendall’s Tau correlation analysis (for variable assignment and data, see Supplementary Table 2). Correlations with MIR are framed in black. Kendall’s Tau values are shown from red to blue as indicated in the legend. Source data are provided in the Source Data file. MIR minimum infection rate, mosq mosquito, ab abundance, hum humidity, cov coverage, div diversity, imperv imperviousness, hum pop dens human population density, seq sequence, temp temperature. **b** Linear regression (conditional means and 95% confidence intervals) showing differences in bird model predictions and bird observations obtained from field surveys at the five study sites in 2025 (see Supplementary Methods). The dashed black line refers to a 1:1 representation of observed vs. modelled species abundances, and the points represent the raw data per species and site.

Hierarchical cluster analysis suggested that WNV infection in mosquitoes was primarily correlated with spatial proximity and water availability rather than mosquito community composition. C and RA clustered together, as did S and N, with RB linking closer to S and N (Fig. 5b, d). RB and N lacked large water sources, while S contained a small constructed rainwater retention site but no higher trees. Correlation analyses were performed using field variables (e.g. mosquito abundance, bloodmeal sources, MIR, temperature and humidity), modelled bird abundances of WNV susceptible species^62,63^, as well as microhabitat characteristics (e.g. impervious surface and human population density). Mosquito MIR in 2023 was positively correlated with open water, as well as with the abundance of the great tit (*Parus major*) and the common wood pigeon (*Columba palumbus*) (Fig. 6a, Supplementary Table 2). Correlations between mosquito MIR and avian species dataset were less significant for 2024 (Supplementary Table 3, Supplementary Fig. 5) and for combined analyses of 2023 and 2024 (Supplementary Fig. 6), most likely due to lower WNV infection rates. The modelled bird abundances were confirmed by a breeding bird field survey at the study sites in the spring of 2025 (Supplementary Figs. 2–4, Supplementary Methods). Bird abundances were well correlated for all sites, except for S and N, where the recorded bird abundance was lower than in our model, which for S could be due to little vegetation cover (Fig. 6b).

Modelled abundances of *Parus major* and *Cyanistes caeruleus*, and bird host richness inferred from blood fed mosquitoes had the highest representation (cos^2^) in 2023 whereas bush cover, minimum temperature and presence of DNA from *Sylvia atricapilla* in mosquito bloodmeals contributed little (Supplementary Fig. 7). In 2024, several bird species detected in mosquito bloodmeals and mosquito richness had highest cos^2^ values (Supplementary Fig. 8a), while mosquito abundance, presence of *Erithacus rubecula* in bloodmeals and bird diversity had highest representations in analyses of the 2023 and 2024 datasets (Supplementary Fig. 9a).

**Fig. 7:**
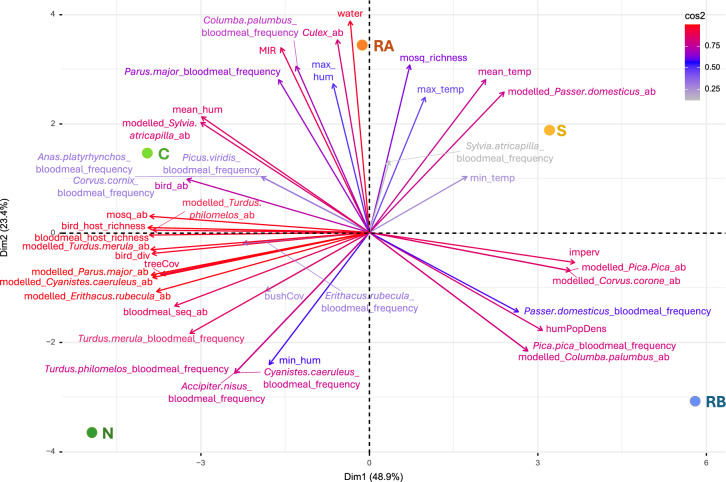
Principle component analysis (PCA) of variables associated with mosquito MIR in 2023. The biplot of the PCA linked mosquito MIR to the abundances of the bird species based on bloodmeal host frequency in the field sampling and bird species abundance (based on a spatial model), as well as with spatial characteristics of the sites (tree cover, imperviousness, presence of water, human density). Modelled positions of the study sites in the biplot are marked by coloured dots. Source data are provided in the Source Data file. MIR minimum infection rate, mosq mosquito, ab abundance, hum humidity, Cov coverage, div diversity, imperv imperviousness, humPopDens human population density, seq sequence, temp temperature.

Principal Component Analysis (PCA) revealed that the first two principal components (PCs) explained 72.3 % of data variability in 2023 (Fig. 7). PC1, reflecting a gradient from urban to more natural and biodiverse habitats, explained 48.9 % of the variance, while PC2, related to climatic factors, accounted for 23.4%. Mosquito MIR was positively associated with *Culex* sp. abundance, with frequencies of the great tit (*Parus major*), the common wood pigeon (*Columba palumbus*) in mosquito bloodmeals, as well as with the presence of permanent larger water bodies, mean to maximum humidity, and semi-natural habitats. MIR was negatively correlated with the detection of the Eurasian magpie (*Pica pica*) and the house sparrow (*Passer domesticus*) in mosquito bloodmeals, the modelled abundance of the wood pigeon (*Columba palumbus*), human population density and imperviousness, indicating that the two most contrasting habitats, urbanised and highly natural, biodiverse areas, were linked to lower mosquito infection rates.

In 2024, as well as in combined analyses for both years, mosquito MIR was also associated with open water bodies, *Culex* sp. abundance and a diverse avian feeding pattern of mosquitoes (bird host richness) in semi-natural areas (Supplementary Figs. 7b and 9b). Urbanisation variables, such as imperviousness and human population density, remained negatively associated, as did the Eurasian magpie (*Pica pica*) and the carrion crow (*Corvus corone*), while certain passerine species, such as the Eurasian blackcap (*Sylvia atricapilla*) and the Eurasian chaffinch (*Fringilla coelebs*), were positively correlated with mosquito MIR. (Supplementary Table 4). Overall, these findings reveal that microhabitat structure defines local host composition and seems to drive fine-scale WNV amplification in an urban area.

## Discussion

Urban areas are potential hotspots for mosquito-borne diseases, particularly those transmitted by *Aedes aegypti* and *Aedes albopictus*, such as dengue, chikungunya and Zika virus, which circulate between humans and mosquitoes^64^. Although humans are dead-end hosts for WNV, urban areas are often affected. Climate change and urbanisation are key drivers of increasing mosquito-borne disease incidence^65^. Understanding how urban microenvironments facilitate transmission is critical for designing healthy, climate-resilient infrastructures and for identifying urban areas with elevated WNV transmission risk to guide public awareness campaigns and vector control.

In this study, we analysed WNV infection rates in mosquitoes at a fine spatial scale across five closely situated urban sites, each less than 1 km apart, in Berlin during the 2023 and 2024 transmission seasons. We observed high rates of WNV infection in mosquitoes (up to 4.8%), with local amplification strongly influenced by urban land cover features. Greener sites with abundant bushy vegetation, water bodies and trees, specifically a park-like residential area and cemetery, exhibited the highest MIRs (up to 20.8), whereas a residential backyard with little vegetation showed low rates (up to 2.2). Of note, despite high tree and bush cover, a nature conservation site showed significantly lower WNV infection rates throughout both seasons (MIRs up to 4.0), and a sponge city site with little vegetation showed a similar, but less pronounced pattern. These findings highlight the importance of small-scale surveillance to identify urban WNV hotspots.

Genomic surveillance and phylogenetic analyses revealed that the vast majority of detected WNV sequences clustered within the Berlin clade of WNV lineage 2^55^, confirming local endemicity and suggesting that observed mosquito infections resulted from local amplification rather than repeated introductions. The extensive local amplification and maintenance of closely related variants over consecutive years support the importance of microhabitat-level ecological conditions and suggest more limited virus dispersal via infected birds in urban settings. In contrast, higher rates of dispersal of related virus variants have been observed in mosquitoes from rural areas of eastern Germany^66^. Five WNV strains clustered within the broader genetic diversity of detected WNV sequences in Germany and were most likely introduced from other regions in the country. These sequences exhibited between 30 and 94 SNVs and were substantially more diverse than the 62 locally amplified strains, which showed 0–31 SNVs. We found no evidence of subsequent local amplification of these introduced strains. Variation in WNV infection rates between sites is therefore likely driven by urban environmental characteristics rather than by virus introductions.

While higher mosquito densities can correlate with increased WNV infection risk^31^, our data indicate that abundance alone does not explain the local infection patterns. RA and C showed both high mosquito density and high MIRs in July and August, whereas low-density sites sometimes exhibited elevated infection rates (e.g. RB in July; RA and C in September 2023). Notably, the nature conservation site had in both years the highest mosquito numbers, but the lowest WNV infection rates. Differences in species assemblages, such as low frequency of competent vectors, could contribute to these patterns, as observed elsewhere^67^. However, we detected no significant variation in mosquito species composition or diversity between sites, consistent with other studies^68^. The majority of mosquitoes at all sites were *Cx. pipiens* (>82%) and 92.7% belonged to the genus *Culex*, the principal vector for WNV^69^. *Aedes vexans*, a potential WNV vector, showed no significant site differences and has conflicting evidence regarding its vector competence^70,71^.

Mosquito infection depends on feeding on viraemic birds, and urban characteristics influence bird abundance and community composition^28–30,63^, which in turn affect WNV infection rates. Data on bird species abundance and species assemblages were not available for our study sites in the mosquito sampling years, but bird abundances were modelled based on environmental descriptor variables and breeding bird censuses^63^, and confirmed by a bird field survey conducted at the study sites in 2025. Analysis of bird species assemblages and mosquito bloodmeals suggested that the common wood pigeon (*Columba palumbus*), the great tit (*Parus major*), the hooded crow (*Corvus cornix*), the Eurasian blackcap (*Sylvia atricapilla*), the Eurasian chaffinch (*Fringilla coelebs*) and the green woodpecker (*Picus viridis*) were associated with higher mosquito infection rates. Of note, the bird field survey showed that common species, such as great tits (*Parus major*), were underpredicted in the model, especially at S and N. The sites RA and C, which had the highest WNV infection rates, also had the highest bird abundances, driven by the most common species, such as great tits (*Parus major*) and wood pigeons (*Columba palumbus*). These species were mostly detected in mosquito bloodmeals at C and RA, supporting the hypothesis that these common species are involved in WNV amplification.

*Culex* mosquitoes feed predominantly on birds, with occasional human hosts. Different bird species contributed to blood meals at RB and RA, which may explain higher WNV infection rates at RA due to a higher proportion of potential avian amplificatory hosts^30^. Conversely, low WNV infection rates at N may reflect higher bird diversity with more incompetent or less competent hosts^28,29^. Differences in host assemblages may also explain the contrasting patterns at C, which exhibited both higher host diversity and elevated MIRs.

Urban green infrastructure modulates environmental conditions such as temperature and humidity and is important for preserving urban biodiversity^72^. In Berlin, greener urban areas correspond to higher bird diversity^63,73,74^. We observed significantly lower WNV infection rates at the nature conservation and sponge city sites, supporting the hypothesis that higher biodiversity reduces infection risk, as suggested by PCA results. The sponge city site showed a more than ten-fold increase in mosquito infection rates from July (0.97) to August (12.70) in 2023, likely reflecting heavy rainfall, standing water in retention basins and elevated temperatures that accelerated mosquito development.

Observed MIRs of this study were relatively high in 2023 and comparable to European regions with recurring outbreaks and high numbers of human cases, e.g. Northern Italy and Central Greece^59–61^. This contrasts with the reporting of a single human WNV case in Berlin in 2023 and seven in 2024, suggesting underdiagnosis due to no or mild symptoms upon infection and low public and physician awareness, as well as the predominantly avian feeding behaviour of *Cx pipiens*. This species occurs in two bioforms^75^, with biotype *pipiens* feeding preferentially on birds and biotype *molestus* on humans, with hybrids discussed to act as bridge vectors for bird-to-human transmission^76^, even though recent studies have also contradicted this hypothesis^77,78^. Our blood-meal analyses provide evidence for occasional human exposure, and two autochthonous WNND cases were reported in proximity to our study sites in 2021 and 2022^55^. The high density and infection rates of WNV vectors at RA, with a substantial elderly population as the highest risk group for severe disease, highlight the public health relevance of these findings^23^.

This study has some constraints that should be considered when interpreting the findings. Sampling was restricted to a single area comprising five microhabitats, and it remains to be determined whether similar patterns occur in other parts of the Berlin metropolitan area that include comparable microhabitat structures. Although consistent trends were observed across two consecutive sampling seasons, suggesting microhabitat-specific effects, confirmation in additional urban regions is required to assess generalisability. In addition, the number of blood-fed mosquitoes available for analysis was limited. More comprehensive inference regarding host-vector interactions would benefit from larger numbers of blood-fed specimens collected throughout the mosquito season and across microhabitats. Future studies integrating blood meal analyses with simultaneous bird community assessments and systematic testing of birds for WNV infection at the same sites would clarify which avian species contribute most to local WNV amplification and help disentangle microhabitat-specific transmission dynamics.

In conclusion, these data demonstrate that urban landscape features, host community composition and biodiversity significantly influence WNV amplification at fine spatial scales. Identifying urban hotspots provides opportunities for spatially targeted surveillance, control of *Culex* larvae by reducing breeding sites, and public awareness campaigns, similar to approaches used for *Aedes*-borne infections^9–13,73,74^. Similar methodologies are needed for *Culex*-borne infections, particularly in light of the emergence of WNV in Europe and the goals of implementing climate-resilient cities and nature-based solutions in urban planning.

## Methods

### Site selection, mosquito collection and identification

The study area is located in Berlin, the largest and most populous metropolitan area in Germany, with an area of 892 km^2^ and a population of 3.8 million (Fig. 1). The following five mosquito trapping sites were selected representing a gradient from green to grey infrastructure, with or without additional open water provisioning: (1) a plain backyard of a Berlin-typical residential block in a grey urban space, hereafter called RB, constructed in 1930, multiple dwelling house with two blocks for 600–700 inhabitants and a backyard with a lawn of 5000 m^2^ (sampling permission and access granted by the private proprietor); (2) a residential area (RA) built in 1918 according to the garden city concept, which comprises a park-like property with old trees and a glacial pond, and consists of 163 multiple dwelling and 69 single-family houses for ca. 2500 inhabitants (sampling permission and access granted by the private proprietor and inhabitants); (3) a large park-like cemetery (C) with a glacial pond, established in 1908, area of 110,342 m^2^ (sampling permission granted by the Bezirksamt Tempelhof-Schöneberg von Berlin at 2023-06-12 (AG/24_2023) and at 2024-06-05 (AG/24_2024)); (4) a nature-based solution site, an industrial site reconstructed according to the sponge city concept (S), re-constructed 2010–2015, area of 43,368 m^2^, unsealed 23,974 m^2^, containing a rainwater basin reservoir and a rain garden, a cistern, a flood retention lake, permeable pavement, a natural garden, green roofs, infiltration systems, evaporation areas, 83 renting parties (mainly companies with offices and workshops) (sampling permission and access granted by the private proprietor); and (5) a nature conservation area (N), established in 1999, a re-natured railway yard, area of 180,000 m^2^, containing a highly diverse flora and fauna (sampling permission granted by the Senatsverwaltung für Mobilität, Verkehr, Klimaschutz und Umwelt von Berlin at 2023-06-20 and 2024-06-13 (OA-AS/FAS/836)). Overview maps from Germany and Berlin districts (Fig. 1a) were created using QGIS 3.4.12 software (Open Source Geospatial Foundation; http://qgis.org; 2025) incorporating map data from OpenStreetMap (https://www.openstreetmap.org/copyright; 2025) and digital geographic data from the German Federal Office for Cartography and Geodesy (Bundesamt für Kartographie und Geodäsie; https://gdz.bkg.bund.de/index.php/default/digitale-geodaten/verwaltungsgebiete/nuts-gebiete-1-5-000-000-stand-31-12-nuts5000-31-12.html; 2024) and Berlin (https://daten.odis-berlin.de/de/dataset/bezirksgrenzen/; 2024). The detailed satellite image from the sampling area (Fig. 1b) was adapted from an aerial picture from 2023 published by the Berlin geoportal (https://gdi.berlin.de/viewer/main/; accessed 2026-04-15).

Mosquitoes were trapped with four or three BG-Pro traps (Biogents, Germany) per site, baited with BG-Mozzibait (Biogents, Germany) and dry ice as the CO_2_-source for four consecutive nights (from dusk until dawn) per month in the mosquito season from June to September in 2023 or 2024, respectively. Traps were placed at fixed positions approximately 20-50 m apart, except at RA, where one spot was changed in July 2023 due to accessibility. Temperature and humidity were measured at each of the mosquito collection sites using thermo-/hygrometers (Temperature & Humidity Logger DS1923-F5#, Maxim Integrated, USA; see Source Data). Mosquitoes were morphologically identified on ice using a standard key^20^.

The diversity of mosquito species for each site was calculated using the Shannon diversity index^79^: *H* = −∑pi*ln(*pi*), with *pi* defined as the number of individuals of one species divided by the total number of individuals collected per site. Species evenness, a measure of the relative abundance of the different species (*S*) per site, was calculated as *E* = *H*’/ln(*S*).

### RNA extraction, molecular species identification and WNV screening

In total, we pooled 1-10 individual specimens according to species, gender and location, before homogenisation in ice-cold 500 µL phosphate-buffered saline (PBS) using ceramic beads at 30 Hz for 2 × 50 s in a TissueLyser II (Qiagen, Germany). Superpools of 10 pools with 100 µL homogenate of each pool were prepared for further analyses. Genomic nucleic acids (NA) were either extracted from 200 µL of the superpools or from 50 µL of the pool homogenate and eluted in a total volume of 100 µL using the MagNA Pure 96 DNA and Viral NA Small Volume Kit (Roche Diagnostics, Germany).

For single specimens, the mosquito species was confirmed by partial sequencing the mosquito cytochrome c oxidase I (COI) gene using 1 µL NA and a published protocol^80^. In brief, a 710 bp fragment of the mosquito COI gene was amplified using primers LCO1490 (5′- GGTCAACAAATCATAAAGATATTGG-3′) and HCO2198 (5′-TAAACTTCAGGGTGACCAAAAAATCA-3′) using Platinum Taq Polymerase (Thermo Fisher Scientific, USA) and the subsequent thermal protocol: 3 min at 94 °C, followed by 35 cycles of 94 °C for 15 s, 50 °C for 20 s and 72 °C for 40 s and a final elongation step at 72 °C for 5 min. PCR amplicons were treated with ExoSAP (Biotechrabbit, Germany) according to the manufacturer’s instructions before Sanger sequencing by Microsynth Seqlab (Microsynth AG, Switzerland). Sequences were analysed using the BLASTn web tool (NCBI, USA).

For cDNA synthesis, eluted NA from mosquito superpools or pool homogenates was transcribed to cDNA using SuperScript IV reverse transcriptase (Thermo Fisher Scientific GmbH, Germany) and random hexamer primers (Integrated DNA Technologies Germany GmbH, Germany). Screening for the presence of WNV genomic RNA was performed using 2 µL cDNA by a published real-time PCR (RT-PCR) assay^81^. In brief, amplification of a fragment of the WNV genome was performed using primers FLI-WNV5-F (5′-GGGCCTTCTGGTCGTGTTC-3′) and FLI-WNF6-R (5′-GATCTTGGCYGTCCACCTC-3′) and the Platinum Taq Polymerase (Thermo Fisher Scientific, USA) with the subsequent thermal protocol: 2 min at 95 °C, followed by 45 cycles of 95 °C for 15 s and 60 °C for 30 s. Real-time measurement of WNV genome amplification was performed using the FLI-WNF-Probe (5′-FAM/CCACCCAGG/ZEN/AGGTCCTTCGCAA/3IABkFQ-3′) and the LightCycler LC480 (Roche Diagnostics, Germany). WNV-positive pool homogenates were confirmed by at least one of the following PCRs: (1) a nested- PCR amplifying WNV ORF nt 2049–2910 (forward: 5′-TGGGAAGAGGAGAACAGCAGAT-3′, reverse: 5′-GCGGTTCCTATGATCTTCGAGTC-3′; forward nested: 5′-CAGGGACTTCTAGGAGCCCT-3′, reverse nested: 5′-TCAGGAACATGCGAGTGCTT-3′), (2) a nested- PCR amplifying WNV ORF nt 7633-8488 (forward: 5′-AGGGGAAGTTTGGAAGGAGAGA-3′, reverse: 5′-TGGTAGTTCCAGGTCCTGTAGG-3′; forward nested: 5′-TCAACCACATGACGAAGGAAG-3′, reverse nested: 5′-GTTGCGTGAAAGGGGGTTTC-3′), or (3) the commercially available RealStar® WNV RT-PCR Kit (Altona Diagnostics, Germany). Primers were designed using Geneious Prime 9.1.8 (Biomatters, New Zealand) and commercially ordered (Integrated DNA Technologies, Belgium). The conventional PCRs were performed using Platinum Taq Polymerase (Thermo Fisher Scientific, USA) and the subsequent thermal protocol: 3 min at 95 °C, followed by 35 cycles of 95 °C for 15 s, 60 °C for 20 s and 72 °C for 40 s and a final elongation step at 72 °C for 5 min. After treatment with ExoSAP (Biotechrabbit, Germany), PCR amplicons were Sanger sequenced by Microsynth Seqlab (Microsynth AG, Switzerland) and analysed using BLASTn.

### Blood meal analysis of mosquitoes

Mosquito pools containing blood-fed mosquitoes were tested for the host species source by a vertebrate COI PCR^82^ (*n* = 199) using 1 µl eluted NA. Vertebrate COI fragments were amplified using primers Mod_RepCOI_F (5′-TNTTYTCMACYAACCACAAAGA-3′), VertCOI_7216_R (5′-CARAAGCTYATGTTRTTYATDCG-3′), VertCOI_7194_F (5′-CGMATRAAYAAYATRAGCTTCTGAY-3′) and Mod_repCOI_R (5′-TTCDGGRTGNCCRAARAATCA-3′) using Platinum Taq Polymerase (Thermo Fisher Scientific, USA) and the subsequent thermal protocol: 3 min at 95 °C, followed by 40 cycles of 95 °C for 40 s, 48.5 °C for 30 s and 72 °C for 30 s and a final elongation step at 72 °C for 7 min. Amplicons were sequenced as described above and analysed using BOLD v4^83^ and BLASTn. Visualisation was done using SankeyMATIC (https://www.sankeymatic.com; accessed 2026-02-13).

### WNV whole genome sequencing and phylogenetic analyses

For sequencing of the complete coding regions of detected WNV sequences, native and amplicon-based next-generation sequencing (NGS) were performed. For native NGS, libraries were prepared from 5 µL RNA of WNV-positive mosquito homogenates using the KAPA RNA Hyper Prep Kit (Roche Diagnostics, Germany), quantified using the Qubit dsDNA HS Assay kit (Thermo Fisher Scientific, USA) and the Agilent TapeStation with the HS D1000 Kit (Agilent, Germany), and applied to an Illumina NextSeq 2000 platform (Illumina, USA).

For amplicon-based NGS, WNV genomic sequences were amplified using a multiplex PCR described before^66^. In brief, two PCR sets amplifying the WNV open reading frame were performed using Platinum Taq Polymerase (Thermo Fisher Scientific, USA) and the subsequent thermal protocol: 3 min at 95 °C, followed by 40 cycles of 95 °C for 15 s, 60 °C for 15 s and 72 °C for 30 s, and a final elongation step at 72 °C for 5 min, and merged for subsequent NGS analysis. Library preparation was performed using the KAPA Hyper Plus Kit (Roche Diagnostics), quantified as described for native NGS, and amplicon-based NGS was performed using Illumina MiSeq v3, 600 cycles, for the samples from 2023 and Illumina NextSeq 2000 for the samples from 2024.

WNV genomes were assembled using Geneious Prime 9.1.8 (Biomatters, New Zealand). Reads were trimmed using BBDuk (v35.82 by Brian Bushnell) and mapped to the German WNV reference sequence OP810567. The coding-complete regions of detected WNV sequences as well as publicly available reference sequences from Germany (NCBI virus, USA) were aligned using MAFFT v7.308^84^. Following a model test with MEGA v11.0.13, a maximum likelihood (PHYML) tree was inferred using the IQ-TREE web server^85,86^, employing the TN93 substitution model and 1000 bootstrap replicates with reference sequence AY532665 as an outgroup. Visualisation was performed using the Interactive Tree of Life (iTOL) web server^87^.

### Biodiversity analyses

We performed a Kendall Tau’s correlation analysis and a principal component analysis (PCA) on a dataset of biotic and abiotic variables across the five sampling locations to reduce dimensionality and identify key variables explaining most of the variability in the data and to understand their contribution to WNV infections in mosquitoes. We extracted environmental variables in a 50 m radius around the focal location (i.e. the location in the centre around the four trap positions) and calculated the mean of the variable values falling within the radius per location: availability of open water (binary; yes or no), imperviousness [%], tree cover [%], human population density [no. inhabitants/ha], and bush cover [%]. These data were downloaded from the Berlin geoportal (publicly available at: https://gdi.berlin.de; accessed 2025-02-07; see Source Data). Additionally, we used the mean, minimum and maximum temperatures and humidities measured during the sampling occasions as climatic descriptors (Supplementary Tables 2–4).

As biotic variables, we included the bird and mosquito community composition and abundance. Mosquito community composition and abundance were obtained from the field (Table 1), and unidentified species were discarded. Rarefaction to standardise mosquito diversity was done with the R-package iNext^88^ (Supplementary Fig. 1). Bird community information was obtained from a joint species distribution model in a hierarchical Bayesian framework (jSDM)^89,90^. The model calculated breeding bird diversity and total abundance of 66 non-aquatic breeding birds as well as abundance of single species in Berlin, as projected to a raster of 100 × 100 m resolution^62,63^. The original data for the model consisted of counts of breeding territories in 29 transects across the city; the fitted jSDM related bird presence and abundance to environmental predictors (tree cover, human population density, open green space, noise level), as well as prey species abundances (insects and spiders), which were based on a jSDM using distance to water as a predictor variable^63^. To confirm the modelled bird species at the study sites, we conducted an intensive field survey in 2025 (Supplementary Methods, Supplementary Figs. 2–4). Similar to above, we extracted the modelled total bird diversity and total abundance in a 50 m radius around the location, as well as the predicted presence and abundance of the single bird species. This yielded a total of 42 bird species present and their abundances around the sampling locations (Supplementary Table 5). In addition, the frequencies of host feeding on susceptible bird species detected in the mosquito bloodmeal analyses were included as variables in the Kendall’s tau correlation. Susceptible bird species were defined as species in which WNV RNA or antibodies were detected in previous studies^91–93^.

### Statistical analysis

For normalisation over the mosquito seasons, mosquito numbers per trap per night were calculated. *P*-values for statistically significant differences in the normalised mosquito numbers were calculated with a two-tailed unpaired Welch’s *t*-test comparing single trap performance per night for each site and month. The MIR assumes that at least one mosquito per pool is positive. MIR was calculated as the number of positive pools divided by the total number of individual mosquitoes tested, expressed as the minimum number infected per 1000. The *p*-values for statistical significance of the MIRs per site were calculated with a two-way Chi-Squared test (MedCalc Software Ltd., Belgium). Detailed test statistics for the *t*- and Chi-Squared tests can be found in the Source Data.

We conducted a cluster analysis using the R-package pheatmap (Kolde R, 2025. pheatmap: Pretty Heatmaps. R package version 1.0.13, https://CRAN.R-project.org/package=pheatmap) for the mosquito abundances at the five locations, as well as the infection probability relative to the total abundance of mosquitoes at that location, as a preliminary check whether infections follow mosquito community composition. Data were standardised prior to PCA to ensure the contribution of all variables was comparable. The number of components was determined using the Kaiser criterion, retaining those with eigenvalues > 1. The analysis was conducted using the function *prcomp()*. We used cosine squared (cos²) values to measure the proportion of the variable’s total variance that is explained by a specific dimension, also called ‘quality of representation’. High values close to 1 mean the variable was well-represented by the dimension and that most of the variable’s variability was captured by that component. Low values close to zero mean poor representation of a component, and its variability is spread across other dimensions. We used the R-package ‘factoextra’ for visualisation^94^.

### Reporting summary

Further information on research design is available in the Nature Portfolio Reporting Summary linked to this article.

## Supplementary information

Supplementary Information

Peer Review file

Reporting Summary

## Source data

Source Data

## Data Availability

The data generated in this study are provided in the Source Data file. WNV sequence datas are available under GenBank accession numbers numbers PX569566.1–PX569613.1 and PZ018997.1–PZ019015.1 (WNV complete ORFs; https://www.ncbi.nlm.nih.gov/nuccore/PX569566.1 to https://www.ncbi.nlm.nih.gov/nuccore/PZ019015.1) as well as PX620687.1–PX620726.1 and PZ019016.1–PZ019020.1 (WNV partial sequences; https://www.ncbi.nlm.nih.gov/nuccore/PX620687.1 to https://www.ncbi.nlm.nih.gov/nuccore/PZ019020.1); NGS data under BioSample accessions SAMN53337887–SAMN53337935 and SAMN55391934–SAMN55391951 (BioProject ID PRJNA1367344; https://www.ncbi.nlm.nih.gov/biosample/1367344). Source data are provided with this paper.

## Code availability

Analysis code and data are available under 10.5281/zenodo.19577441.
