# Supplementary material for: Fine-scale heterogeneity and local amplification of West Nile virus in urban environments in Berlin
Source: Nat Commun. 2026 Jun 12;17:4597. doi: 10.1038/s41467-026-73251-5 (PMC13263328; doi:10.1038/s41467-026-73251-5)
Supplement: Supplementary file 1 — Supplementary Information [file 41467_2026_73251_MOESM1_ESM.pdf]

Supplementary Information on manuscript:

Fine-scale heterogeneity and local amplification of West Nile virus in urban environments in Berlin

Corinna Patzina-Mehling, Anne Kopp, Yea-Seul Lee, Maximillian X.L. Birkl, Sophia Ebers, Katrin Voigt, Selina L. Graff, Uli Beisel, Conny Landgraf, Florian Ganz, Aimara Planillo, Stephanie Kramer-Schadt, Sandra Junglen

Table of contents:

Supplementary Tables

Supplementary Table 1……………………………………………………………………………………..2

Supplementary Table 2……………………………………………………………………………………..3

Supplementary Table 3……………………………………………………………………………………..4

Supplementary Table 4……………………………………………………………………………………..5

Supplementary Table 5……………………………………………………………………………………..6

Supplementary Figures

Supplementary Figure 1……………………………………………………………………………………7

Supplementary Figure 2……………………………………………………………………………………8

Supplementary Figure 3……………………………………………………………………………………9

Supplementary Figure 4……………………………………………………………………………………10

Supplementary Figure 5……………………………………………………………………………………11

Supplementary Figure 6……………………………………………………………………………………12

Supplementary Figure 7……………………………………………………………………………………13

Supplementary Figure 8……………………………………………………………………………………14

Supplementary Figure 9……………………………………………………………………………………15

Supplementary Methods

Field survey execution……………………………………………………………………………………..16

Field survey data preparation and statistical analysis…………………………………………….16

Field survey results…………………………………………………………………………………………16

Supplementary References…………………………………………………………………………………………..17

Supplementary Tables

Supplementary Table 1: Mosquito diversity per site per month for each sampling year. Species richness, Shannon diversity index and evenness are depicted.

		June			July			August			September			Total		
	Sampling year	Species richness	Shannon index	Evenness	Species richness	Shannon index	Evenness	Species richness	Shannon index	Evenness	Species richness	Shannon index	Evenness	Species richness	Shannon index	Evenness
RB	2023	5	0.4696	0.2918	4	0.4223	0.3046	7	0.9505	0.4885	2	0.4966	0.7165	10	0.5646	0.2452
	2024	5	0.6997	0.4347	7	0.2471	0.1270	5	0.5841	0.3629	1	0.0000	0.0000	9	0.4885	0.2223
RA	2023	8	0.3283	0.1579	9	0.2591	0.1179	10	0.6210	0.2697	4	0.2755	0.1987	12	0.3641	0.1465
	2024	8	0.2699	0.1298	8	0.2578	0.1240	10	0.3806	0.1653	6	0.4612	0.2574	14	0.3148	0.1193
C	2023	6	0.2770	0.1546	11	0.3551	0.1481	8	0.5265	0.2532	7	0.9819	0.5046	11	0.4283	0.1786
	2024	8	0.5235	0.2518	8	0.5819	0.2799	8	0.8947	0.4303	6	0.7710	0.4303	12	0.7342	0.2955
S	2023	4	0.2137	0.1542	10	0.3247	0.1410	5	0.4386	0.2725	2	0.2706	0.3904	10	0.3538	0.1536
	2024	7	0.6749	0.3468	5	0.1929	0.1199	7	0.3167	0.1628	2	0.2019	0.2913	10	0.3481	0.1512
N	2023	7	0.4549	0.2338	8	0.2912	0.1401	8	0.5778	0.2779	5	0.6159	0.3827	10	0.4516	0.1961
	2024	9	0.7151	0.3254	9	0.2445	0.1113	10	0.7868	0.3417	4	0.4839	0.3490	13	0.4969	0.1937

Supplementary Table 2: Environmental, climatic and bird abundance variables per site for 2023. Climatic data was collected during sampling and MEAN, MAX and MIN values were calculated. Bird frequencies were based on the bloodmeal analysis or the modelled bird species abundance data.

#	Site [a]	RB	RA	C	S	N
1	mosq_richness	10	12	10	11	10
2	mosq_ab	669	2624	3927	2205	4202
3	Culex_ab	0.92	0.95	0.95	0.96	0.93
4	bird_div	19	27	32	25	37
5	bird_ab	52	76	132	72	91
6	humPopDens	270.76	38.50	3.96	23.85	0
7	water	0	1	1	1	0
8	treeCov	12.10	29.71	71.39	1.03	67.79
9	bushCov	9	37.25	0	0	48
10	imperv	82	15	3	85	9
11	max_temp	26.3	30.0	25.8	29.9	27.1
12	min_temp	15.49	15.20	15.57	15.39	14.99
13	mean_temp	19.95	20.38	19.76	20.23	19.71
14	max_hum	92.93	98.48	93.94	95.20	94.72
15	min_hum	46.98	42.09	49.13	42.09	47.67
16	mean_hum	73.82	77.89	76.74	75.73	76.92
17	MIR	1.49	9.53	7.89	4.08	2.86
18	bloodmeal-seq_ab	11	15	16	8	21
19	bloodmeal-host richness	2	6	11	5	11
20	bird-host richness	2	5	8	4	8
21	*Accipiter-gentilis*_bloodmeal_frequency	0	0	0	0	0
22	*Accipiter-nisus*_bloodmeal_frequency	0	0	0	0	1
23	*Anas-platyrhynchos*_bloodmeal_frequency	0	0	1	0	0
24	*Ardea-cinerea*_bloodmeal_frequency	0	0	0	0	0
25	Modelled-*Columba-livia*_ab	0	0	0	0	0
26	*Columba-livia*_bloodmeal_frequency	0	0	0	0	0
27	Modelled-*Columba-palumbus*_ab	5	4	4	4	4
28	*Columba-palumbus*_bloodmeal_frequency	0	6	3	1	1
29	Modelled-*Corvus-corone*_ab	2	1	1	2	1
30	*Corvus-cornix*_bloodmeal_frequency	0	0	1	0	0
31	*Curruca-curruca*_bloodmeal_frequency	0	0	0	0	0
32	Modelled-*Cyanistes-caeruleus*_ab	5	8	10	5	12
33	*Cyanistes-caeruleus*_bloodmeal_frequency	0	0	0	0	2
34	*Emberiza-calandra*_bloodmeal_frequency	0	0	0	0	0
35	Modelled-*Erithacus-rubecula*_ab	1	4	6	1	9
36	*Erithacus-rubecula*_bloodmeal_frequency	0	0	1	1	1
37	Modelled_*Fringilla-coelebs*_ab	0	0	0	0	0
38	*Fringilla-coelebs*_bloodmeal frequency	0	0	0	0	0
39	Modelled-*Parus-major*_ab	6	8	10	6	11
40	*Parus-major*_bloodmeal_frequency	0	1	1	0	0
41	Modelled-*Passer-domesticus*_ab	15	15	15	15	14
42	*Passer-domesticus*_bloodmeal_frequency	9	4	0	0	1
43	Modelled-*Pica-pica*_ab	1	0	0	1	0
44	*Pica-pica*_bloodmeal_frequency	2	0	0	0	0
45	*Picus-viridis*_bloodmeal_frequency	0	0	1	0	0
46	Modelled-*Sylvia-atricapilla*_ab	2	3	4	3	3
47	*Sylvia-atricapilla*_bloodmeal_frequency	0	1	0	2	1
48	Modelled-*Turdus-merula*_ab	8	9	10	8	10
49	*Turdus-merula*_bloodmeal_frequency	0	1	2	1	6
50	Modelled-*Turdus-philomelos*_ab	0	2	3	0	3
51	*Turdus-philomelos*_bloodmeal_frequency	0	0	0	0	1

[a] Abbreviations: Cov, coverage; div, diversity; Bloodmeal-seq_ab, number of host detections in mosquito bloodmeals; humPopDens, human population density; mosq, mosquito; ab, abundance; temp, temperature; hum, humidity; MIR, minimum infection rate; imperv, imperviousness; water, presence of larger open water bodies.

Supplementary Table 3: Environmental, climatic and bird abundance variables per site for 2024. Climatic data was collected during sampling and MEAN, MAX and MIN values were calculated. Bird frequencies were based on the bloodmeal analysis or the modelled bird species abundance data.

#	Site [a]	RB	RA	C	S	N
1	mosq_richness	9	14	12	10	13
2	mosq_ab	777	2616	2208	1574	3288
3	Culex_ab	0.88	0.96	0.83	0.95	0.90
4	bird_div	19	27	32	25	37
5	bird_ab	52	76	132	72	91
6	humPopDens	270.76	38.50	3.96	23.85	0
7	water	0	1	1	1	0
8	treeCov	12.10	29.71	71.39	1.03	67.79
9	bushCov	9	37.25	0	0	48
10	imperv	82	15	3	85	9
11	max_temp	28.10	29.66	26.29	26.35	26.15
12	min_temp	16.41	16.16	15.91	15.78	15.26
13	mean_temp	21.13	21.26	20.06	20.17	19.88
14	max_hum	88.99	89.74	91.99	89.70	92.81
15	min_hum	42.96	42.30	50.48	47.03	47.93
16	mean_hum	71.05	72.30	77.34	74.32	71.76
17	MIR	0	5.35	3.17	1.27	0.61
18	bloodmeal-seq_ab	13	14	11	6	19
19	bloodmeal-host richness	4	10	5	4	9
20	bird-host richness	3	9	3	3	7
21	*Accipiter-gentilis*_bloodmeal_frequency	0	0	0	0	1
22	*Accipiter-nisus*_bloodmeal_frequency	0	0	0	0	1
23	*Anas-platyrhynchos*_bloodmeal_frequency	0	0	0	0	0
24	*Ardea-cinerea*_bloodmeal_frequency	0	1	0	0	0
25	Modelled-*Columba-livia*_ab	1	1	0	1	0
26	*Columba-livia*_bloodmeal_frequency	0	1	0	0	0
27	Modelled-*Columba-palumbus*_ab	5	4	4	4	4
28	*Columba-palumbus*_bloodmeal_frequency	1	1	5	0	4
29	Modelled-*Corvus-corone*_ab	2	1	1	2	1
30	*Corvus-cornix*_bloodmeal_frequency	0	0	0	0	0
31	*Curruca-curruca*_bloodmeal_frequency	1	0	0	0	0
32	Modelled-*Cyanistes-caeruleus*_ab	5	8	10	5	12
33	*Cyanistes-caeruleus*_bloodmeal_frequency	0	0	0	0	0
34	*Emberiza-calandra*_bloodmeal_frequency	0	1	0	0	0
35	Modelled-*Erithacus-rubecula*_ab	1	4	6	1	9
36	*Erithacus-rubecula*_bloodmeal_frequency	0	2	1	0	1
37	Modelled_*Fringilla-coelebs*_ab	1	7	12	1	16
38	*Fringilla-coelebs*_bloodmeal frequency	0	1	0	0	0
39	Modelled-*Parus-major*_ab	6	8	10	6	11
40	*Parus-major*_bloodmeal_frequency	0	0	0	2	3
41	Modelled-*Passer-domesticus*_ab	15	15	15	15	14
42	*Passer-domesticus*_bloodmeal_frequency	7	2	0	0	0
43	Modelled-*Pica-pica*_ab	0	0	0	0	0
44	*Pica-pica*_bloodmeal_frequency	0	0	0	0	0
45	*Picus-viridis*_bloodmeal_frequency	0	0	0	0	0
46	Modelled-*Sylvia-atricapilla*_ab	0	0	0	0	0
47	*Sylvia-atricapilla*_bloodmeal_frequency	0	0	0	0	0
48	Modelled-*Turdus-merula*_ab	8	9	10	8	10
49	*Turdus-merula*_bloodmeal_frequency	0	2	0	2	5
50	Modelled-*Turdus-philomelos*_ab	0	2	3	0	3
51	*Turdus-philomelos*_bloodmeal_frequency	0	0	0	1	2

[a] Abbreviations: Cov, coverage; div, diversity; Bloodmeal-seq_ab, number of host detections in mosquito bloodmeals; humPopDens, human population density; mosq, mosquito; ab, abundance; temp, temperature; hum, humidity; MIR, minimum infection rate; imperv, imperviousness; water, presence of larger open water bodies.

<u>Supplementary Table 4:</u> Environmental, climatic and bird abundance variables per site for 2023 and 2024 combined. Climatic data was collected during sampling and MEAN, MAX and MIN values for August 2023 and 2024 were calculated. Bird frequencies were based on the bloodmeal analysis or the modelled bird species abundance data.

#	Site [a]	RB	RA	C	S	N
1	mosq_richness	11	14	12	13	14
2	Mosq_ab	1446	5240	6135	3779	7490
3	Culex_ab	0.8963	0.9567	0.9046	0.9553	0.9147
4	bird_div	19	27	32	25	37
5	bird_ab	52	76	132	72	91
6	humPopDens	270.76	38.50	3.96	23.85	0
7	water	0	1	1	1	0
8	treeCov	12.10	29.71	71.39	1.03	67.79
9	bushCov	9	37.25	0	0	48
10	Imperv	82	15	3	85	9
11	max_temp_August	33.1	37.1	31.1	35.1	31.5
12	min_temp_August	15.1	14.6	14.6	14.6	14.6
13	mean_temp_August	22.3170	22.9182	21.8415	22.1226	21.5528
14	max_hum_August	97.2	100	97.4	100	100
15	min_hum_August	34.0	27.1	35.6	33.4	37.7
16	mean_hum_August	67.2789	70.9733	70.9398	70.1845	73.2785
17	MIR	0.6916	7.4427	6.1940	2.9108	1.8692
18	Bloodmeal-seq_ab	24	29	27	14	40
19	Bloodmeal-host richness	5	12	11	8	14
20	Bird-host richness	4	11	8	6	10
21	*Accipiter-gentilis*_bloodmeal_frequency	0	0	0	0	1
22	*Accipiter-nisus*_bloodmeal_frequency	0	0	0	0	2
23	*Anas-platyrhynchos*_bloodmeal_frequency	0	1	1	0	0
24	*Ardea-cinerea*_bloodmeal_frequency	0	1	0	0	0
25	Modelled-*Columba-livia*_ab	1	1	0	1	0
26	*Columba-livia*_bloodmeal_frequency	0	1	0	0	0
27	Modelled-*Columba-palumbus*_ab	5	4	4	4	4
28	*Columba-palumbus*_bloodmeal_frequency	1	7	8	1	5
29	Modelled-*Corvus-corone*_ab	2	1	1	2	1
30	*Corvus-cornix*_bloodmeal_frequency	0	0	1	0	0
31	*Curruca-curruca*_bloodmeal_frequency	1	0	0	0	0
32	Modelled-*Cyanistes-caeruleus*_ab	5	8	10	5	12
33	*Cyanistes-caeruleus*_bloodmeal_frequency	0	0	0	0	2
34	*Emberiza-calandra*_bloodmeal_frequency	0	1	0	0	0
35	Modelled-*Erithacus-rubecula*_ab	1	4	6	1	9
36	*Erithacus-rubecula*_bloodmeal_frequency	0	2	2	1	2
37	Modelled_*Fringilla-coelebs*_ab	1	7	12	1	16
38	*Fringilla-coelebs*_bloodmeal frequency	0	1	0	0	0
39	Modelled-*Parus-major*_ab	6	8	10	6	11
40	*Parus-major*_bloodmeal_frequency	0	1	1	2	3
41	Modelled-*Passer-domesticus*_ab	15	15	15	15	14
42	*Passer-domesticus*_bloodmeal_frequency	16	6	0	0	1
43	Modelled-*Pica-pica*_ab	1	0	0	1	0
44	*Pica-pica*_bloodmeal_frequency	2	0	0	0	0
45	*Picus-viridis*_bloodmeal_frequency	0	0	1	0	0
46	Modelled-*Sylvia-atricapilla*_ab	2	3	4	3	3
47	*Sylvia-atricapilla*_bloodmeal_frequency	0	1	0	2	1
48	Modelled-*Turdus-merula*_ab	8	9	10	8	10
49	*Turdus-merula*_bloodmeal_frequency	0	3	2	3	11
50	Modelled-*Turdus-philomelos*_ab	0	2	3	0	3
51	*Turdus-philomelos*_bloodmeal_frequency	0	0	0	1	3

[a] Abbreviations: Cov, coverage; div, diversity; Bloodmeal-seq_ab, number of host detections in mosquito bloodmeals; humPopDens, human population density; mosq, mosquito; ab, abundance; temp, temperature; hum, humidity; MIR, minimum infection rate; imperv, imperviousness; water, presence of larger open water bodies.

<u>Supplementary Table 5:</u> **Predicted bird abundances per site.** The modelled bird species abundance per site is listed. Bird species community composition was used as component explaining variability in the data.

Bird species	RB	RA	C	S	N
Acrocephalus_palustris	0	0	1	0	1
Acrocephalus_scirpaceus	0	0	1	0	0
Aegithalos_caudatus	0	1	1	0	1
Alauda_arvensis	0	1	1	0	1
Apus_apus	2	1	0	1	0
Carduelis_carduelis	0	1	0	1	1
Carduelis_chloris	2	2	1	2	2
Certhia_brachydactyla	1	2	3	1	4
Certhia_familiaris	0	1	1	0	1
Coccothraustes_coccothraustes	0	1	2	0	4
Columba_livia	1	1	0	1	0
Columba_palumbus	5	4	4	4	4
Corvus_corone	2	1	1	2	1
Cyanistes_caeruleus	5	8	10	5	12
Dendrocopos_major	1	3	6	1	8
Emberiza_citrinella	0	0	1	0	0
Emberiza_schoeniclus	0	1	1	0	2
Erithacus_rubecula	1	4	6	1	9
Ficedula_hypoleuca	0	1	1	0	1
Fringilla_coelebs	1	7	12	1	16
Garrulus_glandarius	0	0	1	0	1
Parus_cristatus	0	1	1	0	1
Luscinia_megarhynchos	0	1	1	1	1
Muscicapa_striata	0	0	0	0	1
Parus_major	6	8	10	6	11
Passer_domesticus	15	15	15	15	14
Passer_montanus	1	1	1	2	1
Parus_ater	0	0	1	0	1
Phoenicurus_ochruros	2	1	0	1	0
Phoenicurus_phoenicurus	1	1	1	2	1
Phylloscopus_collybita	0	1	1	1	1
Phylloscopus_sibilatrix	0	2	3	0	4
Pica_pica	1	0	0	1	0
Regulus_ignicapilla	0	1	2	0	3
Serinus_serinus	1	1	0	1	1
Sitta_europaea	1	3	5	1	7
Sturnus_vulgaris	2	3	3	3	3
Sylvia_atricapilla	2	3	4	3	3
Sylvia_curruca	0	0	0	1	0
Troglodytes_troglodytes	1	4	6	1	8
Turdus_merula	8	9	10	8	10
Turdus_philomelos	0	2	3	0	3

Supplementary Figures

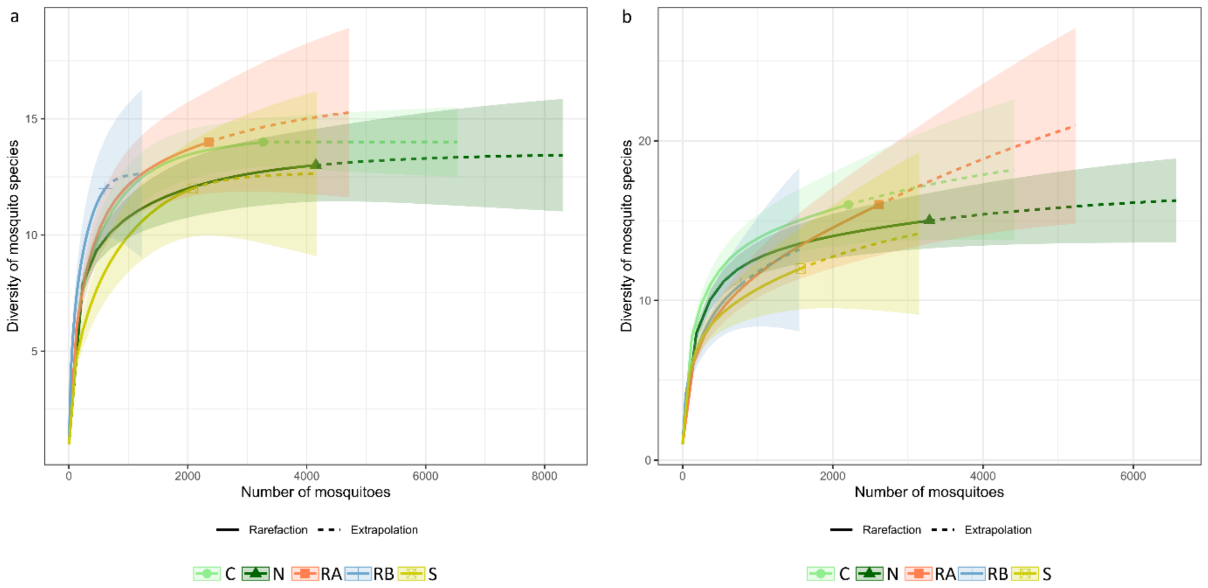

Supplementary Figure 1: Rarefaction plots for mosquito species per site. Rarefaction plots (Hill number q=0) for single mosquito species with unidentified species omitted for 2023 (**a**) and 2024 (**b**).

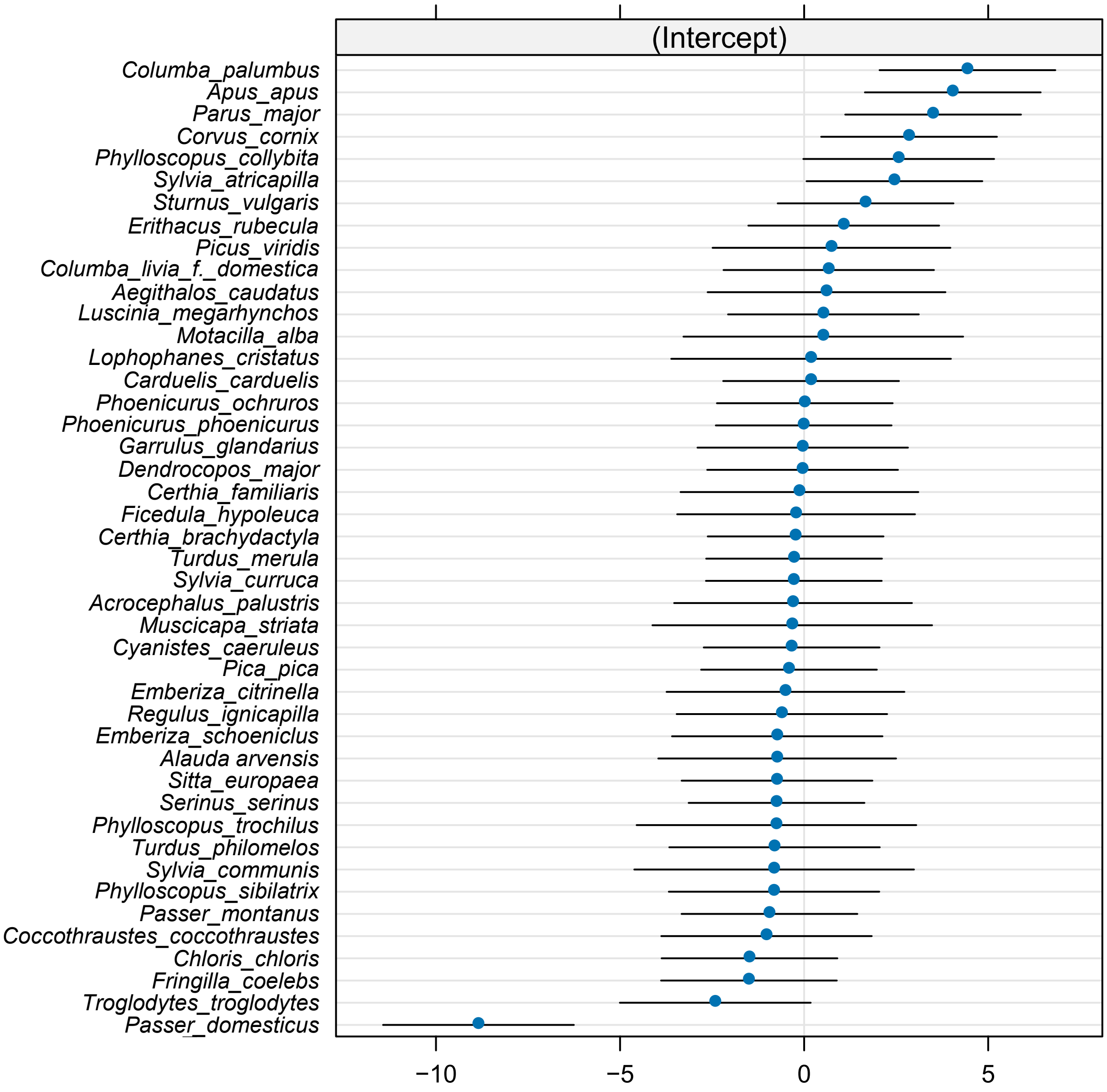

<u>Supplementary Figure 2:</u> Bird abundances in observed versus modelled data. Random effect plot of bird species driving the differences in observed versus modelled abundances (when black error bar exceeds the 0-line). Positive values mean more observations than predicted by the model.

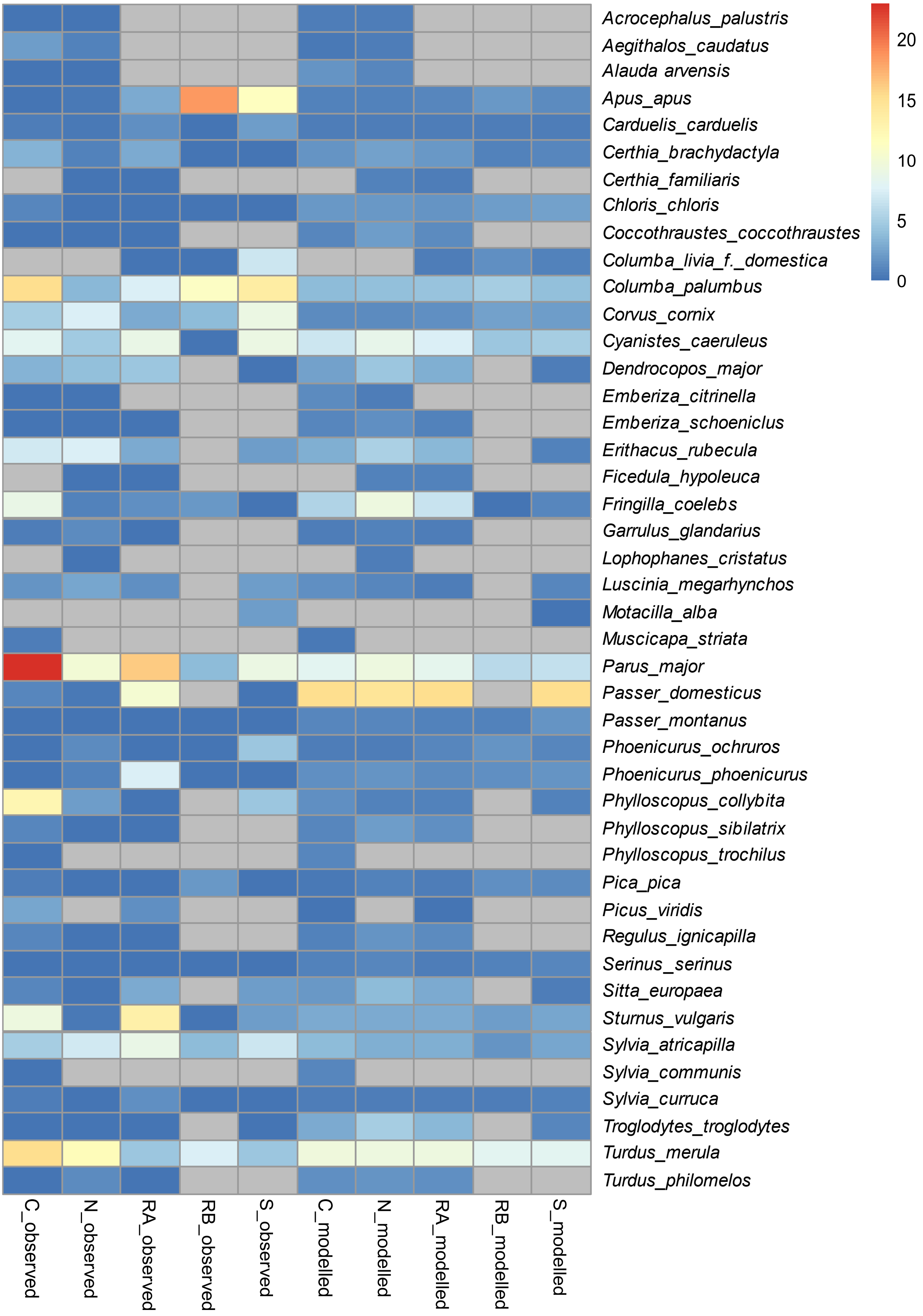

<u>Supplementary Figure 3:</u> Comparison of the observed bird abundances to the modelled data. Heatmap showing the mean bird index values per site observed and modelled. In RB, a huge flock of sparrows (*Passer domesticus*) was removed from the data set. Grey = NA values (species not observed or presence not predicted by the model).

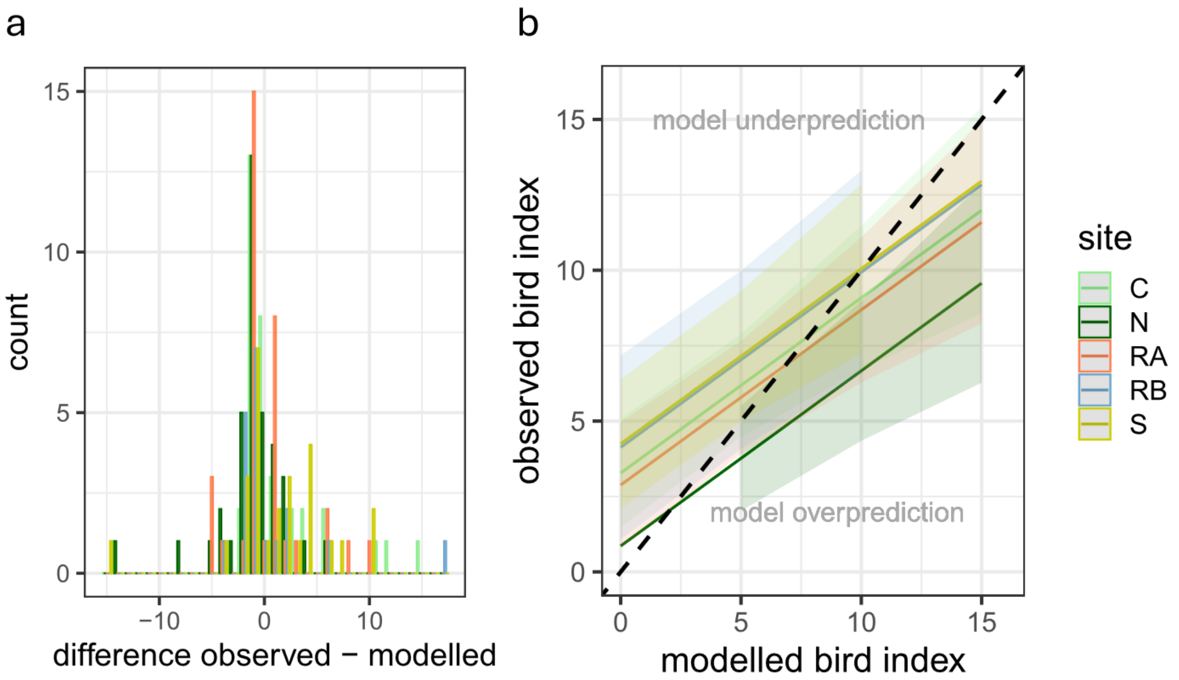

<u>Supplementary Figure 4:</u> Bird field survey differences to bird model data. a Distribution of the differences in the bird indices. The colours refer to the five sites. **b** Conditional plot of zero-inflated linear model for assessing differences in model predictions and observations. The dashed black line refers to a 1:1 representation of observed vs. modelled.

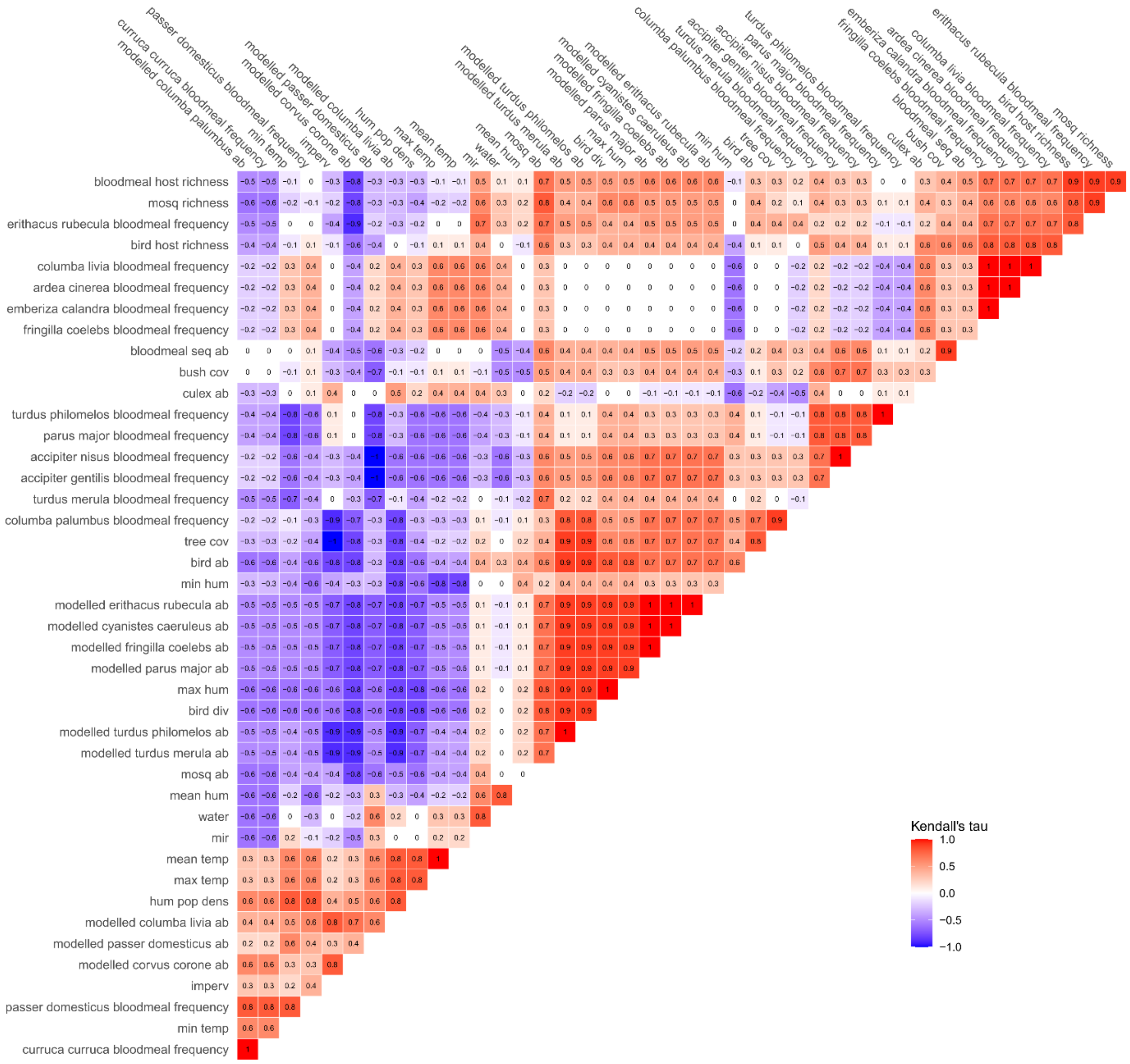

<u>Supplementary Figure 5:</u> Correlation analysis of variables associated with mosquito MIR in 2024. Variables from field sampling in 2024, from a spatial model on local bird abundances, as well as from the environment were used for an extensive Kendall Tau's correlation analysis (for variable assignment and data, see **Supplementary Table 3**).

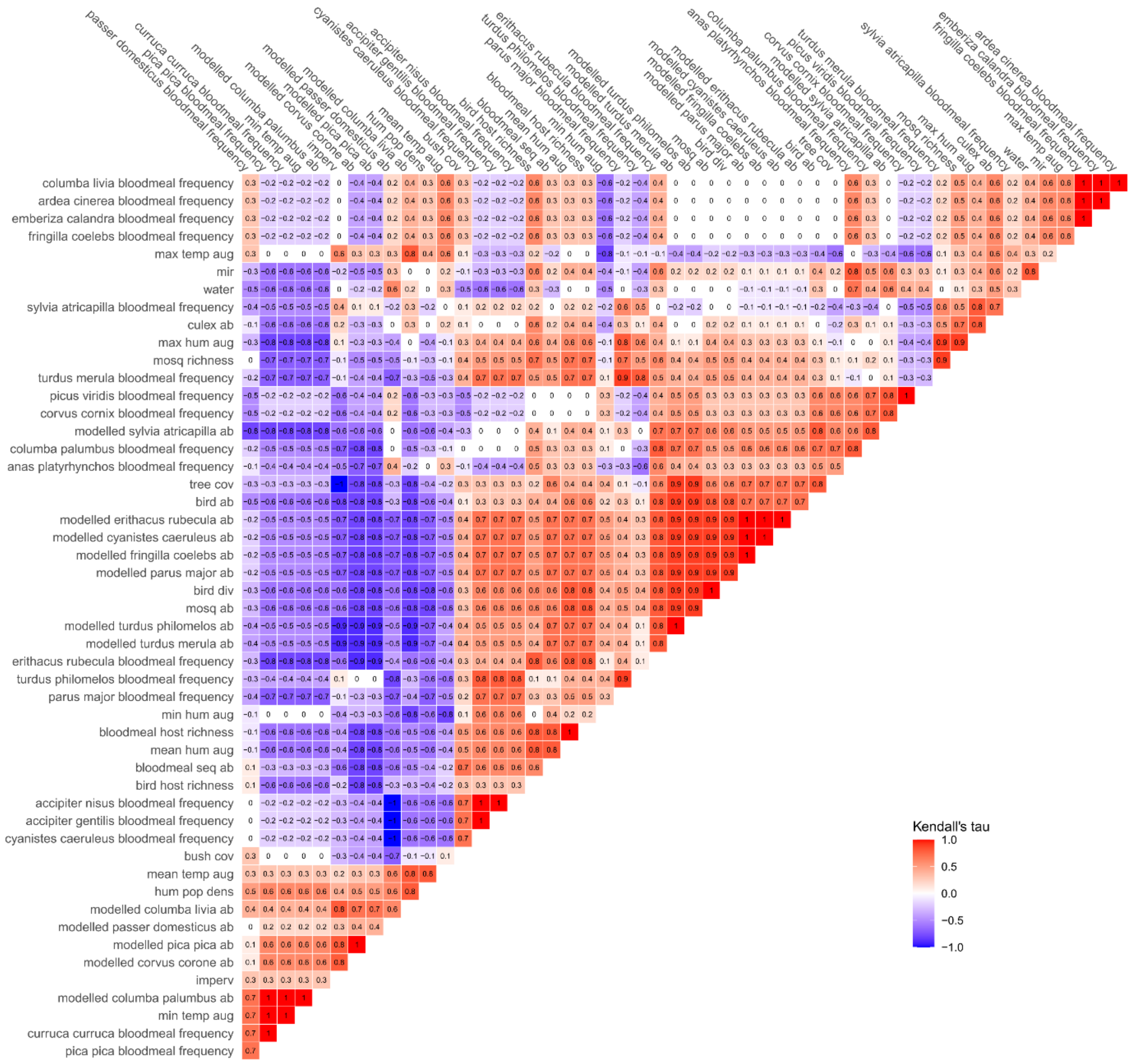

Supplementary Figure 6: Correlation analysis of variables associated with mosquito MIR in 2023 and 2024 combined. Variables from field sampling in 2023 and 2024 combined, from a spatial model on local bird abundances, as well as from the environment were used for an extensive Kendall Tau's correlation analysis (for variable assignment and data, see **Supplementary Table 4**).

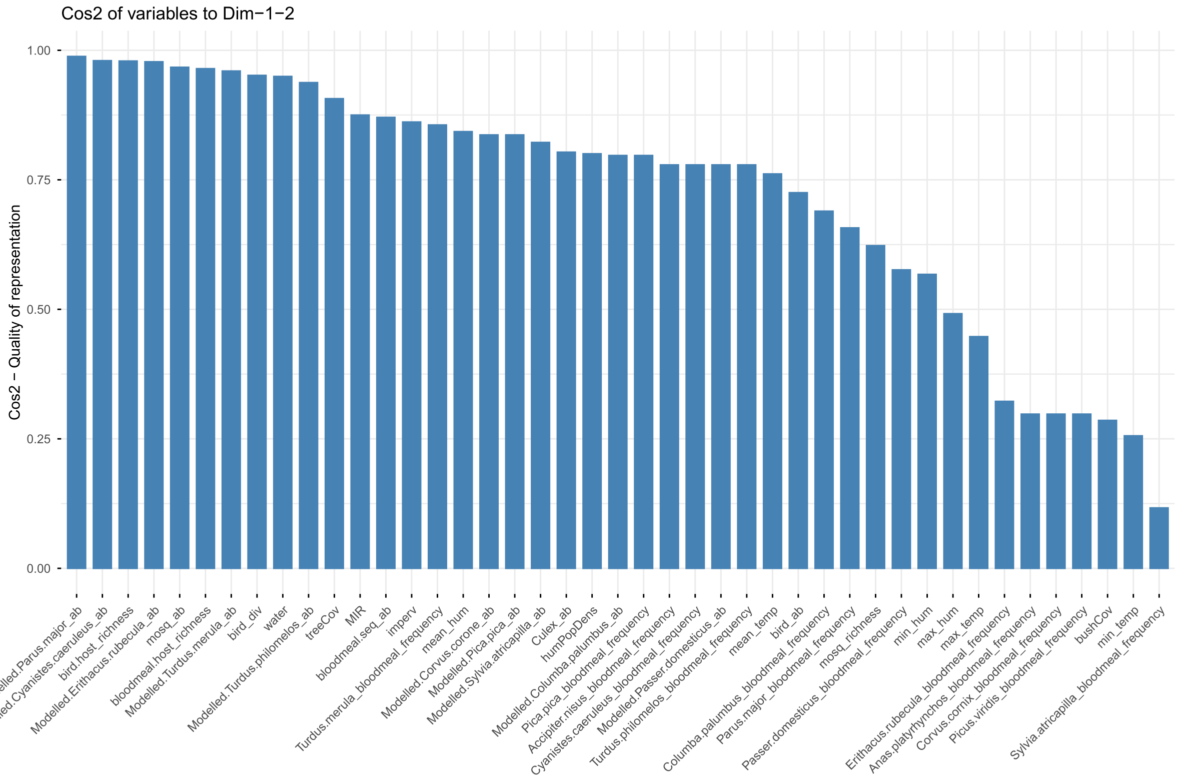

<u>Supplementary Figure 7:</u> Quality of representation of the principal component analysis of the data from 2023 (**Supplementary Table 2**) to determine how much each variable is represented in a given component using square cosine (Cos2). A high value means a good representation of the variable on that component, a low one means imperfect representation.

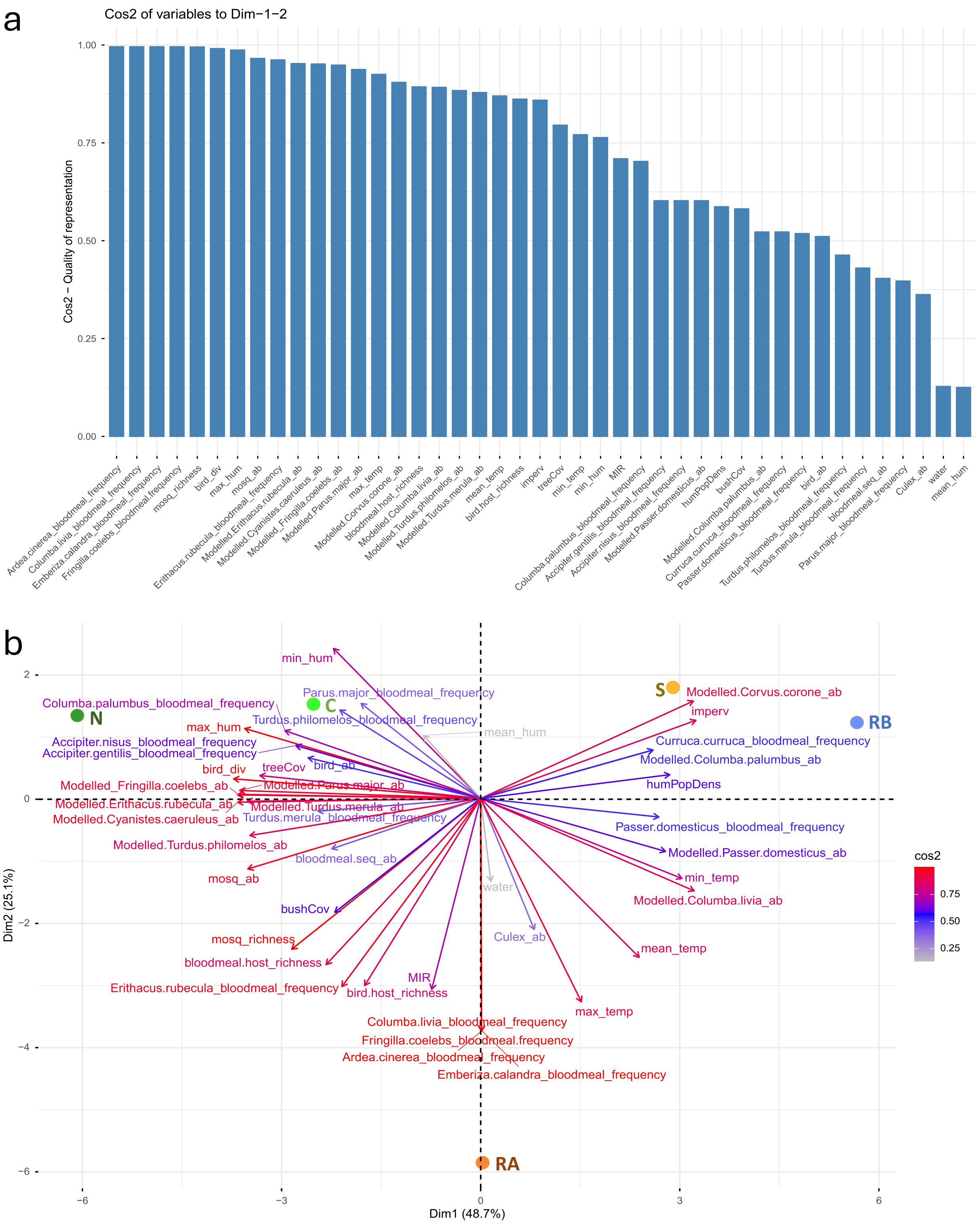

<u>**Supplementary Figure 8:**</u> **Principle component analysis (PCA) of variables associated with mosquito MIR in 2024. a** Quality of representation of the PCA of the data from 2024 (**Supplementary Table 3**) to determine how much each variable is represented in a given component using square cosine (Cos2). A high value means a good representation of the variable on that component, a low one means imperfect representation. **b** The biplot of the PCA linked mosquito MIR to the abundances of the bird species based on bloodmeal host frequency in the field sampling and bird species abundance (based on a spatial model), as well as with spatial characteristics of the sites (tree cover, imperviousness, presence of water, human density). Modelled positions of the study sites in the biplot are marked by coloured dots.

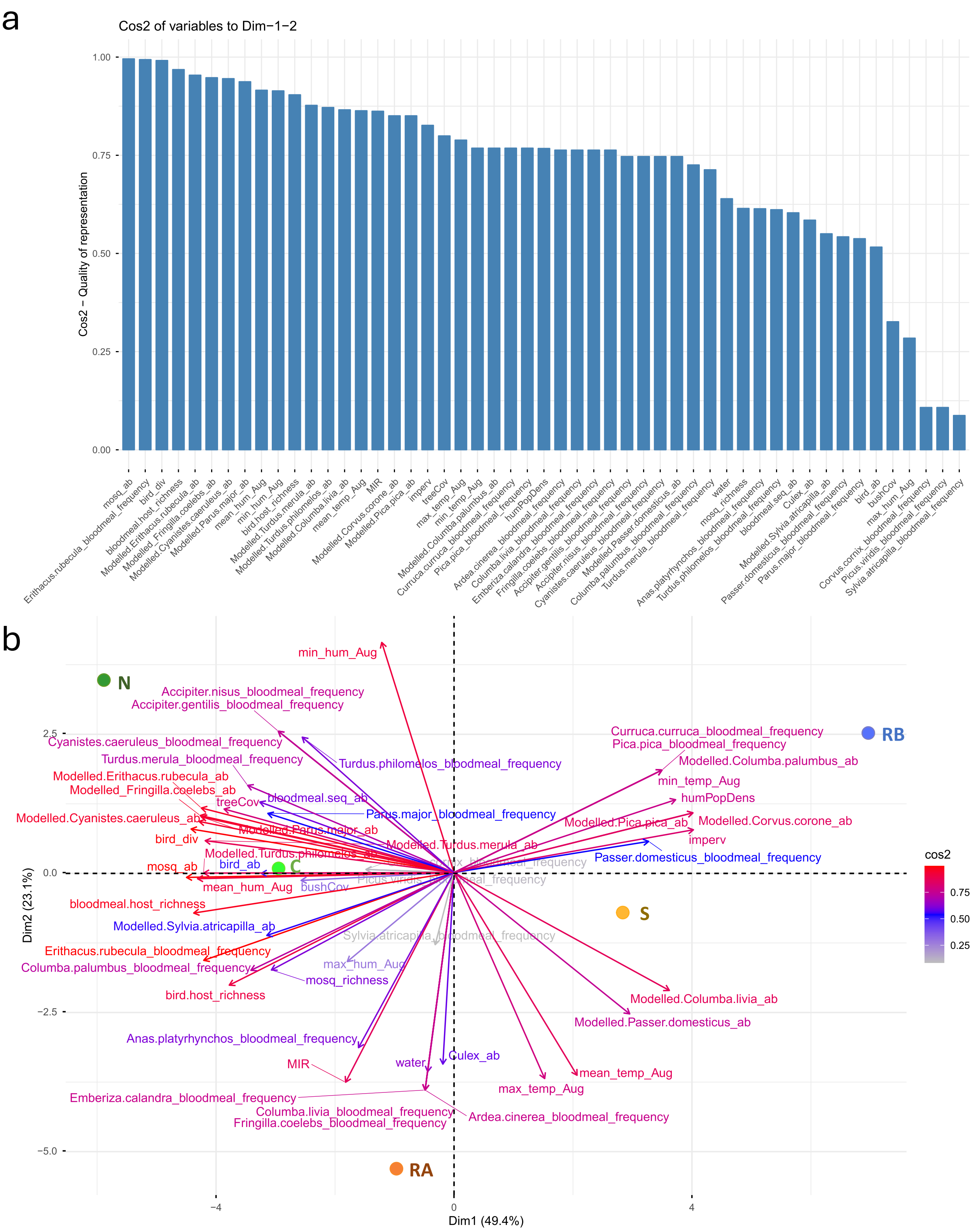

Supplementary Figure 9: Principle component analysis (PCA) of variables associated with mosquito MIR in 2023 and 2024 combined. a Quality of representation of the PCA of the data from 2023 and 2024 combined (**Supplementary Table 4**) to determine how much each variable is represented in a given component using square cosine (Cos2). A high value means a good representation of the variable on that component, a low one means imperfect representation. **b** The biplot of the PCA linked mosquito MIR to the abundances of the bird species based on bloodmeal host frequency in the field sampling and bird species abundance (based on a spatial model), as well as with spatial characteristics of the sites (tree cover, imperviousness, presence of water, human density). Modelled positions of the study sites in the biplot are marked by coloured dots. **b**

Supplementary Methods

Field survey execution:

According to the field surveys underlying the Berlin urban bird diversity and abundance model[1], the repeated bird survey also followed the German national standard for breeding bird monitoring[2]. This standardised framework defines protocols for spatially complete breeding bird surveys and provides guidance on survey timing, visit frequency, mapping precision, and breeding evidence classification.

We conducted independent field surveys at the five locations of the study area between 2025-04-08 and 2025-05-30, to validate the modelled bird diversity and abundance. According to Südbeck et al.[2], each site was systematically walked during the early morning hours from half an hour before sunrise up to three and a half hours after sunrise along transects that covered the entire area. Each site was visited four times to account for temporal variation in detectability among species, to allow for reliable territory delineation and to make it comparable to the transect surveys conducted in Planillo et al.[1]. During each visit, all birds observed or heard within the site boundaries were recorded. Behavioural observations such as singing males, courtship displays, nest-building, alarm calls, or feeding of fledglings were also recorded. Based on these behaviours, all individuals recorded during the breeding season were assigned a breeding evidence code following the DDA (Dachverband Deutscher Avifaunisten; German Ornithological Society) classification, which distinguishes between possible, probable, and confirmed breeding.

Field data were collected digitally using QField 3.5.4 – Fangorn, a mobile GIS platform that allowed direct mapping of observations with real-time georeferencing. Each detection was annotated with date of observation, number of individuals counted in one location and breeding evidence codes.

Field survey data preparation and statistical analysis:

All analyses were done using R 4.4.3 (R Core Team 2025; https://www.R-project.org/). According to Planillo et al.[1], the maximum number of individuals per bird species of a single survey out of the four surveys was retained and divided by the transect length to obtain an index of relative bird abundance per km and site surveyed. Likewise, the modelled bird index was obtained by extracting and averaging the modelled value along the transects surveyed at each site. We focused on the 66 bird species modelled in Planillo et al.[1], i.e. we did not consider bird species that were detected during the survey but not previously modelled, such as water birds. Further, we focused on common bird species and rounded the bird index, so that bird indices below 0.5 were set to zero (analogous to the data preparation for the Principal Component Analysis of modelled bird species, infection rates and environmental predictors; main manuscript). Next, we excluded bird species that had zero rounded bird indices both in the observations as well as in the model in all sites. This reduced the dataset to 44 bird species. In one site (RB), a huge flock of sparrows (*Passer domesticus*) was observed as the maximum individuals observed during one survey. To avoid potential bias from this data point, we excluded it from the dataset.

For statistical analysis, differences (diff) in observed versus modelled bird index values per species were calculated as observed minus modelled, i.e. positive values show higher individual abundances and vice versa. We regressed observed bird indices versus the modelled bird indices and the sampling site (5 levels: C, N, RA, RB, S) as additive terms using a zero-inflated linear model with Gaussian error distribution in the R-package glmmTMB[3]. Model diagnostics were done with the R-package DHARMa[4]. Differences in the bird species per site were assessed fitting a linear mixed effects model including site as covariate and species name as random effect.

Field survey results:

The differences in bird indices were not normally distributed (Shapiro-Wilk normality test, $W = 0.85$, $p < 0.001$). Differences were on average around one individual (median = -0.5), but with deviations up to ~15 individuals,

depending on the site (**Supplementary Figure 4a**). The zero-inflated regression explained 66 % of the variance of the modelled bird abundances around the sites (R-package performance[5]). Model diagnostics did not show overdispersion. The zero-inflation term of the model was marginally significant (p = 0.02; see Table below).

Table: Estimates of the zero inflated linear model regressing modelled bird abundance indices for the five sites versus observed abundances.

**Conditional model**				
**Variable**	**Estimate**	**Std. error**	**z-value**	**p-value**
Intercept (based on site C)	3.3	0.9	3.6	< 0.01
Modelled abundance	0.6	0.1	4.5	< 0.01
N	-2.4	1.2	-2.0	< 0.05
RA	-0.4	1.3	-0.3	0.8
RB	0.8	1.7	0.5	0.6
S	1.0	1.3	0.7	0.5
**Zero-inflation model**				
Intercept	-0.4	0.2	-2.3	< 0.05

Residual degrees of freedom: 150; n = 158

On average, the observed bird abundance was well represented by the model, however, the nature park site (N) was under-predicted for higher modelled bird abundances; that is, we observed a lower number of birds than predicted by the model (**Supplementary Figure 4a,b**). Differences were mainly driven by common bird species. The model significantly overestimated bird species appearing in flocks like *Passer domesticus*, while underestimating pigeons (*Columba palumbus*), great tits (*Parus major*) – especially at the cemetery (C), crows (*Corvus cornix*), Eurasian blackcap (*Sylvia atricapilla*), common chiffchaff (*Phylloscopus collybita*) and swifts (*Apus apus*) – especially at the residential block (RB), while the latter are not relevant for bird species coming into close contact with humans, as their lifestyle is basically aerial (**Supplementary Figure 2**).

In general, the cemetery had the highest observed abundances, especially for the above-mentioned species, but also for blackbirds (*Turdus merula*), European robins (*Erithacus rubecula*), common chaffinchs (*Fringilla coelebs*), blue tits (*Cyanistes caeruleus*) and European starlings (*Sturnus vulgaris*) (**Supplementary Figure 2**). Pigeons, blue tits, great tits, sparrows, starlings and blackcaps were also the most observed birds at the residential area (RA) with high West Nile virus infection rates in mosquitoes.

Supplementary References

1 Planillo A, Kramer-Schadt S, Buchholz S, Gras P, von der Lippe M, Radchuk V. Arthropod abundance modulates bird community responses to urbanization. Divers Distrib 2020; 27: 34–49. https://doi.org/10.1111/ddi.13169

2 Südbeck P, Andretzke H, Fischer S, Gedeon K, Pertl C, Linke TJ, Georg M, Koenig C, Schikore T, Schroeder K, Droeschmeister R, Sudfeldt C (2025). Methodenstandards zur Erfassung der Brutvögel Deutschlands. Dachverband Deutscher Avifaunisten e.V. (DDA), Länderarbeitsgemeinschaft der Vogelschutzwarten (LAG VSW) and Bundesamt für Naturschutz (BfN), ISBN: 978-3-9819703-3-3

3 Brooks ME, Kristensen K, van Benthem KJ, Magnusson A, Berg CW, Nielsen A, Skaug HJ, Maechler M, Bolker BM (2017). glmmTMB Balances Speed and Flexibility Among Packages for Zero-inflated Generalized Linear Mixed Modeling. The R Journal, 9(2), 378–400. doi:10.32614/RJ-2017-066

4 Hartig F (2024). DHARMa: Residual Diagnostics for Hierarchical (Multi-Level / Mixed) Regression Models. R package version 0.4.7, https://CRAN.R-project.org/package=DHARMa

5 Lüdecke D, Ben-Shachar MS, Patil I, Waggoner P, Makowski D (2021). performance: An R Package for Assessment, Comparison and Testing of Statistical Models. Journal of Open Source Software, 6(60), 3139. https://doi.org/10.21105/joss.03139
