# Supplementary material for: Fine-scale heterogeneity and local amplification of West Nile virus in urban environments in Berlin
Source: Nat Commun. 2026 Jun 12;17:4597. doi: 10.1038/s41467-026-73251-5 (PMC13263328; doi:10.1038/s41467-026-73251-5)
Supplement: Supplementary file 3 — Reporting Summary [file 41467_2026_73251_MOESM3_ESM.pdf]

nature portfolio

Corresponding author(s): Prof. S. Junglen

Last updated by author(s): Apr 14, 2026

Reporting Summary

Nature Portfolio wishes to improve the reproducibility of the work that we publish. This form provides structure for consistency and transparency in reporting. For further information on Nature Portfolio policies, see our Editorial Policies and the Editorial Policy Checklist.

Statistics

For all statistical analyses, confirm that the following items are present in the figure legend, table legend, main text, or Methods section.

n/a	Confirmed	
☐	☒	The exact sample size (*n*) for each experimental group/condition, given as a discrete number and unit of measurement
☐	☒	A statement on whether measurements were taken from distinct samples or whether the same sample was measured repeatedly
☐	☒	The statistical test(s) used AND whether they are one- or two-sided<br>*Only common tests should be described solely by name; describe more complex techniques in the Methods section.*
☐	☒	A description of all covariates tested
☐	☒	A description of any assumptions or corrections, such as tests of normality and adjustment for multiple comparisons
☐	☒	A full description of the statistical parameters including central tendency (e.g. means) or other basic estimates (e.g. regression coefficient) AND variation (e.g. standard deviation) or associated estimates of uncertainty (e.g. confidence intervals)
☐	☒	For null hypothesis testing, the test statistic (e.g. F, t, r) with confidence intervals, effect sizes, degrees of freedom and P value noted<br>*Give P values as exact values whenever suitable.*
☒	☐	For Bayesian analysis, information on the choice of priors and Markov chain Monte Carlo settings
☒	☐	For hierarchical and complex designs, identification of the appropriate level for tests and full reporting of outcomes
☒	☐	Estimates of effect sizes (e.g. Cohen's *d*, Pearson's *r*), indicating how they were calculated

Our web collection on statistics for biologists contains articles on many of the points above.

Software and code

Policy information about availability of computer code

Data collection	Environmental variables for the correlation analysis were downloaded from the publicly available Berlin geoportal [https://stadtentwicklung.berlin.de/geoinformation/fis-broker/]. Bird community composition data were extracted from previous studies cited in the Methods section (Planillo et al., 2021a; Planillo et al., 2021b).
Data analysis	QGIS 3.4.12 software (http://qgis.org) incorporating open source map data from OpenStreetMap (https://www.openstreetmap.org/copyright; 2025) and geodata from Germany (https://gdz.bkg.bund.de/index.php/default/digitale-geodaten/verwaltungsgebiete/nuts-gebiete-1-5-000-000-stand-31-12-nuts5000-31-12.html; 2024) and Berlin (https://daten.odis-berlin.de/de/dataset/bezirksgrenzen/; 2024), as well as an aerial picture from 2023 from the Berlin geoportal (https://gdi.berlin.de/viewer/main/; 2026) was used for map generation.<br>PCR amplicons were Sanger sequenced by Microsynth Seqlab (Microsynth AG, Switzerland) and analysed using BOLDv4 and BLASTn (NCBI, USA).<br>Geneious Prime 9.1.8 (Biomatters, New Zealand) was used for primer design and WNV genome assembly using BBDuk (v35.82 by Brian Bushnell) for trimming, MAFFT v7.308 for alignment and PHYML phylogenetic tree inference. MEGA v11.0.13 was used for model testing. IQ-TREE web browser was used to infer the phylogenetic tree and the Interactive Tree of Life (iTOL) web server iTOL was used for visualisation.<br>Statistical analysis was done with Microsoft Excel software (t-test) and MedCalc Software Ltd, Belgium (Chi-Squared test). Bird and mosquito community analyses were conducted with R v 4.4.3 (2025). Examples of R-packages used: Rarefaction was done with the R-package iNext. Cluster analysis was done using the R-package pheatmap, and the R-package 'factoextra' was used for visualisation. Many more R-packages were used for data analysis all specified in the manuscript and code. Analysis code and data are available under [https://doi.org/10.5281/zenodo.19577441].

For manuscripts utilizing custom algorithms or software that are central to the research but not yet described in published literature, software must be made available to editors and reviewers. We strongly encourage code deposition in a community repository (e.g. GitHub). See the Nature Portfolio guidelines for submitting code & software for further information.

Data

Policy information about availability of data

All manuscripts must include a data availability statement. This statement should provide the following information, where applicable:

- Accession codes, unique identifiers, or web links for publicly available datasets
- A description of any restrictions on data availability
- For clinical datasets or third party data, please ensure that the statement adheres to our policy

The data generated in this study are provided in the Source Data file. WNV sequence datas are available under GenBank accession numbers numbers PX569566.1 – PX569613.1 and PZ018997.1 – PZ019015.1 (WNV complete ORFs; https://www.ncbi.nlm.nih.gov/nuccore/PX569566.1 to https://www.ncbi.nlm.nih.gov/nuccore/PZ019015.1) as well as PX620687.1 – PX620726.1 and PZ019016.1 – PZ019020.1 (WNV partial sequences; https://www.ncbi.nlm.nih.gov/nuccore/PX620687.1 to https://www.ncbi.nlm.nih.gov/nuccore/PZ019020.1); NGS data under BioSample accessions SAMN53337887 – SAMN53337935 and SAMN55391934 – SAMN55391951 (BioProject ID PRJNA1367344; https://www.ncbi.nlm.nih.gov/biosample/1367344).

Research involving human participants, their data, or biological material

Policy information about studies with human participants or human data. See also policy information about sex, gender (identity/presentation), and sexual orientation and race, ethnicity and racism.

Reporting on sex and gender	*Use the terms sex (biological attribute) and gender (shaped by social and cultural circumstances) carefully in order to avoid confusing both terms. Indicate if findings apply to only one sex or gender; describe whether sex and gender were considered in study design; whether sex and/or gender was determined based on self-reporting or assigned and methods used. Provide in the source data disaggregated sex and gender data, where this information has been collected, and if consent has been obtained for sharing of individual-level data; provide overall numbers in this Reporting Summary. Please state if this information has not been collected. Report sex- and gender-based analyses where performed, justify reasons for lack of sex- and gender-based analysis.*
Reporting on race, ethnicity, or other socially relevant groupings	*Please specify the socially constructed or socially relevant categorization variable(s) used in your manuscript and explain why they were used. Please note that such variables should not be used as proxies for other socially constructed/relevant variables (for example, race or ethnicity should not be used as a proxy for socioeconomic status). Provide clear definitions of the relevant terms used, how they were provided (by the participants/respondents, the researchers, or third parties), and the method(s) used to classify people into the different categories (e.g. self-report, census or administrative data, social media data, etc.) Please provide details about how you controlled for confounding variables in your analyses.*
Population characteristics	*Describe the covariate-relevant population characteristics of the human research participants (e.g. age, genotypic information, past and current diagnosis and treatment categories). If you filled out the behavioural & social sciences study design questions and have nothing to add here, write "See above."*
Recruitment	*Describe how participants were recruited. Outline any potential self-selection bias or other biases that may be present and how these are likely to impact results.*
Ethics oversight	*Identify the organization(s) that approved the study protocol.*

Note that full information on the approval of the study protocol must also be provided in the manuscript.

Field-specific reporting

Please select the one below that is the best fit for your research. If you are not sure, read the appropriate sections before making your selection.

☐ Life sciences ☐ Behavioural & social sciences ☒ Ecological, evolutionary & environmental sciences

For a reference copy of the document with all sections, see nature.com/documents/nr-reporting-summary-flat.pdf

Ecological, evolutionary & environmental sciences study design

All studies must disclose on these points even when the disclosure is negative.

Study description	Using intensive mosquito sampling within a one-square-kilometer area in Berlin, Germany, for two consecutive mosquito seasons, we examined how urban land cover, including climate-resilient infrastructure, influences local WNV amplification.
Research sample	Blood-seeking adult female mosquitoes were sampled from each site with comparable collection methods. Mosquitoes were identified individually and subsequently pooled according to species, site and date, so that species- and site-specific viral loads could be examined. Blood-seeking female mosquitoes were primarily attracted by the traps due to baiting with CO2 and scent. Crepuscular mosquitoes, such as the West Nile virus (WNV) vector Culex pipiens, were primarily targeted by running the traps from dusk until dawn.
Sampling strategy	Blood-seeking mosquitoes were trapped in a standardised manner using four or three BG-Pro traps per site that were baited with

	CO_2 and scent, for four consecutive nights per month in Berlin, Germany, over the mosquito seasons 2023 or 2024, respectively. Five sites were chosen that represent different urban microhabitats and were less than 1 km apart. Sample size differences according to environmental factors at the different microhabitats were analysed.
Data collection	Temperature and humidity were measured at each of the mosquito collection sites using thermo-/hygrometers (Temperature & Humidity Logger DS1923-F5#, Maxim Integrated, USA). Mosquitoes were collected each morning and identified morphologically, before being homogenised in pools of 10 according to species and site and month. After RNA extraction and cDNA synthesis, specific RT-PCRs were performed for WNV screening.<br>A repeated field survey was conducted following standard monitoring practices to assess breeding bird abundances at the sites, following the German national standard for breeding bird monitoring (Südbeck et al. 2025) and was conducted by Florian Ganz under the supervision of Conny Landgraf. We conducted independent field surveys at the five locations of the study area between 2025-04-08 and 2025-05-30. According to Südbeck et al., each site was systematically walked during the early morning hours from half an hour before sunrise up to three and a half hours after sunrise along transects that covered the entire area. Each site was visited four times to account for temporal variation in detectability among species, to allow for reliable territory delineation. Field data were collected digitally using QField 3.5.4 – Fangorn, a mobile GIS platform that allowed direct mapping of observations with real-time georeferencing. Each detection was annotated with date of observation, number of individuals counted in one location and breeding evidence codes. For details see Supplementary Method.
Timing and spatial scale	Mosquito field sampling was performed for four consecutive nights per month in the mosquito seasons 2023 and 2024. To mainly target crepuscular mosquitoes, such as the West Nile virus (WNV) vector Culex pipiens, the traps were activated from dusk until dawn, and emptied every morning. Sampling was done at following dates: 2023-06-19 until 2023-06-23, 2023-07-17 until 2023-07-21, 2023-08-21 until 2023-08-25, 2023-09-11 until 2023-09-15, 2024-06-17 until 2024-06-21, 2024-07-15 until 2024-07-19, 2024-08-12 until 2024-08-16 and 2024-09-09 until 2024-09-13.<br>Five sites within 1 km distance to each other were selected according to different urban green microhabitat structures. Sampling for each site was performed with four traps in 2023 and three traps in 2024 to minimise trapping site-specific variations.
Data exclusions	When viral load was too low to obtain full genome ORFs, partial sequence information was excluded from phylogenetic analyses.<br>For the rarefaction analysis, unidentified species were excluded. Data on 19th of June were excluded in the 2023 data set since they were missing in one location to keep sampling effort constant.<br>For the correlation analysis comprising both years, only temperature and humidity data from August were included in the analysis, since data from the other months was incomplete.
Reproducibility	For reproducibility, we set up four or three traps at five different sites for four consecutive nights for four months in 2023 or 2024, respectively. With this set-up, variable weather conditions were minimised, single trap performance variations were reduced and site- and month-specific comparisons enabled. Also, the bird field survey was based on four repeated samplings.
Randomization	This is not relevant to our study, since field sampling was performed to examine possible differences between sites in mosquito abundance, mosquito species composition and arbovirus load. Four and later on three traps per site were installed to minimise single trap performance variation to impact the data analysis.
Blinding	Blinding was not relevant in our study, since mosquito pool analysis was not done site-specifically.

Did the study involve field work? ☒ Yes ☐ No

Field work, collection and transport

Field conditions	Standardised sampling was performed in parallel at five sites within a distance of 1 km to each other at four consecutive nights. Temperature and humidity were measured and inserted as environmental variables into the analysis. Due to the parallel sampling, variation in field conditions at the same sampling time points was low.
Location	Five sampling sites were chosen in a known West Nile virus hotspot region in the city of Berlin, Germany, according to diverse green urban microhabitats and biodiversity. Sites were selected for low spatial distances within a distance of 1 km to each other, to minimise site-specific differences in weather conditions and to enable parallel sampling for reduced sampling variation. The sites were located at following coordinates (latitude, longitude): RB: 52.4618, 13.3704; RA: 52.4569, 13.3619; C: 52.4578, 13.3682; S: 52.4663, 13.3656; N: 52.4578, 13.3682.
Access & import/export	Sample collection was primarily performed by accessing the sites on foot or by bike, thus without disturbing the habitats. As urban sites, pedestrians are common at these sites. For three urban residential and industrial sites (S, RA and RB) permission for the set-up of the traps and access to the area was given by the specific proprietors. For the cemetery (C), permission for our study was granted by the Bezirksamt Tempelhof-Schöneberg von Berlin at 2023-06-12 (AG/24_2023) and at 2024-06-05 (AG/24_2024). For field sampling at the nature conservation area, permission was granted by the Senatsverwaltung für Mobilität, Verkehr, Klimaschutz und Umwelt von Berlin at 2023-06-20 and 2024-06-13 (OA-AS/FAS/836).
Disturbance	Sampling was performed with minimal disturbance of microhabitats, since the traps were set up by single persons walking into the areas and setting up the traps hanging at sturdy branches without disturbing the environment.

Reporting for specific materials, systems and methods

We require information from authors about some types of materials, experimental systems and methods used in many studies. Here, indicate whether each material, system or method listed is relevant to your study. If you are not sure if a list item applies to your research, read the appropriate section before selecting a response.

Materials & experimental systems

n/a	Involved in the study
☒	☐ Antibodies
☒	☐ Eukaryotic cell lines
☒	☐ Palaeontology and archaeology
☐	☒ Animals and other organisms
☒	☐ Clinical data
☒	☐ Dual use research of concern
☒	☐ Plants

Methods

n/a	Involved in the study
☒	☐ ChIP-seq
☒	☐ Flow cytometry
☒	☐ MRI-based neuroimaging

Animals and other research organisms

Policy information about studies involving animals; ARRIVE guidelines recommended for reporting animal research, and Sex and Gender in Research

Laboratory animals	The study did not involve laboratory animals.
Wild animals	Blood-seeking, adult female mosquitoes were caught by BG Pro traps over night. Catch bags with living specimens were collected in the morning, transported into the laboratory, anaesthesised using ethanol fumes and sacrificed for further analysis by freezing them at -80°C.
Reporting on sex	Female blood-seeking mosquitoes were attracted by the traps. Male mosquitoes do not feed on blood and were only rarely trapped and analysed.
Field-collected samples	Field collected mosquitoes were stored at -80°C and identified on dry ice. Homogenisation of up to 10 specimens from the same site, month and species were performed on ice, before RNA extraction, cDNA synthesis and PCR analyses were done. For long-term storage, samples were frozen at -80°C.
Ethics oversight	No ethical approval or guidance were required for mosquito trapping and testing.

Note that full information on the approval of the study protocol must also be provided in the manuscript.

Plants

Seed stocks	*Report on the source of all seed stocks or other plant material used. If applicable, state the seed stock centre and catalogue number. If plant specimens were collected from the field, describe the collection location, date and sampling procedures.*
Novel plant genotypes	*Describe the methods by which all novel plant genotypes were produced. This includes those generated by transgenic approaches, gene editing, chemical/radiation-based mutagenesis and hybridization. For transgenic lines, describe the transformation method, the number of independent lines analyzed and the generation upon which experiments were performed. For gene-edited lines, describe the editor used, the endogenous sequence targeted for editing, the targeting guide RNA sequence (if applicable) and how the editor was applied.*
Authentication	*Describe any authentication procedures for each seed stock used or novel genotype generated. Describe any experiments used to assess the effect of a mutation and, where applicable, how potential secondary effects (e.g. second site T-DNA insertions, mosiacism, off-target gene editing) were examined.*
