# Supplementary material for: Fine-scale heterogeneity and local amplification of West Nile virus in urban environments in Berlin
Source: Nat Commun. 2026 Jun 12;17:4597. doi: 10.1038/s41467-026-73251-5 (PMC13263328; doi:10.1038/s41467-026-73251-5)
Supplement: Supplementary file 2 — Peer Review file [file 41467_2026_73251_MOESM2_ESM.pdf]

nature portfolio

Peer Review File

Fine-scale heterogeneity and local amplification of West Nile virus in urban environments in Berlin

Corresponding Author: Professor Sandra Junglen

This file contains all reviewer reports in order by version, followed by all author rebuttals in order by version.

Version 0:

Reviewer comments:

Reviewer #1

(Remarks to the Author)
The authors present here a timely and interesting study, on the fine scale effects of urban land cover on mosquito and bird populations and the effects on WNV ecology. This provides much needed data on possible effects of climate adaptive measures on arbovirus ecology. What has been lacking are high quality studies that do not only focus on mosquitoes (although they are also relatively scarce, especially in the context of climate change adaptation) also incorporate changes in wild bird diversity and abundance and subsequent effects on arbovirus outbreak risks. The authors show clear differences in WNV presence between urban microenvironments in Berlin, in 2023. The manuscript is clearly written and provides high quality, in-depth analyses of virological, weather and ecological data. However, a major drawback of the study is the fact that the study only describes data collected in 2023 and a relative small number of study sites. Incidence of WNV in 2024 in Berlin was also high. The study would significantly improve if 2024 data could also be added and/or if the correlations with bird biodiversity could be substantiated.

Some more detailed suggestions and questions:
Line 88: Here would be good to mention human responses to drought that can also increase vector abundance. Examples are irrigation practices and water storage (eg Hydrologic Conditions Describe West Nile Virus Risk in Colorado)
Line 165-168: “Mosquito MIR was
positively correlated with open water and the abundance of the great tit (Parus major) and the common wood pigeon (Columba palumbus), as inferred from bloodmeal DNA” . Some of this has also remained unclear in other parts of the manuscript: 1) did the predicted bird abundance correlate with results of blood meal analyses? 2) your sample size of bloodmeals is low – how reliable is this correlation?
Line 203-204 Previous literature seems to suggest much more intermixing of strains and less focal presence of WNV. Can you address that in this paragraph?
Line 231-232: what is not completely clear to me is whether you indeed also see higher bird diversity in the included green areas in this specific study?
Line 240: Culex bioforms – include current literature showing that the distinction between biotypes is not so clear
Line 249: effectivity of vector control?
Line 360-362: you base your selection of positive species on 3 papers, while the body of evidence of WNV exposure and infections in Europe is much larger. Did you apply a cutoff, or include all species positive at least once? IN literature there is evidence of at least 200 European species that have tested WNV positive – that may have impacted your analyses and results?
I am missing a legend for figure 6

Reviewer #2

(Remarks to the Author)
Thank you for the opportunity to review your manuscript titled: “Urban land cover drives fine-scale heterogeneity and extensive local amplification of West Nile virus”. Overall, it is a great paper and I want to congratulate the authors on excellent research. I think in the manuscript there are a few limitations missing, two that jumped out to me were the fact that you only looked at one summer and the potential sampling bias of your sites (would the results have changed a lot if you changed sites).

Below are some minor corrections.

Abstract:
Line 19: In some areas climate change and extreme events are actually decreasing mosquito-borne diseases, so perhaps it is more accurate to say, “In some areas climate change is….”

Introduction:
Line 88: Might be good just to give a very brief explanation of what you mean by a “sponge city”
Line 89: I think Nat Comms uses British, rather than American English, so it should be “decentralised”

I then jumped to your methods first.

Methods:
Line 256: “which is with 892 km” is the “with” a typo? So the sentence would be” The study area is located in Berlin, which is 892 km2 and has a population of 3.8 million, the largest and most populous metropolitan area in Germany.” The “has” was also missing.
Line 272: Are you missing a “the” before “CO2-source”?
Line 293: Switch out “analyzed” for “analysed”
Line 342: Remove “in the”, in the sentence, "variable values in the falling within…”

Data availability:
Line 393: Was the Berlin geoportal data also publicly available, and is there a code repo?

Back to the results.

Results:
Line 99: Swap out “standardized” for “standardised”
Line 106: You say that WNV in the mosquitoes was “unexpectedly high”, but do we have a good idea of what the infection rate is in Berlin mosquitoes? Why is this unexpected and did you have a baseline that you were comparing this to?

Discussion:
Line 185: I would also add “Aedes albopictus” too.
Line 187: Switch out “urbanization” with “urbanisation”
Line 193: I think here you need to back up this claim of “unexpected” - you need to link this to literature to understand why this would be unexpected, if the mosquito and host are abundant and you get frequent cases of WNV.
Line 239: I would say underdiagnosis is more complex than that, probably with the most likely reason being mild infection and common febrile symptoms.
Line 245: Perhaps it would be useful to state why the high elderly population would matter?

Version 1:

Reviewer comments:

Reviewer #1

(Remarks to the Author)
I hereby congratulate the authors with a great manuscript and study. The addition of bird survey data and 2024 data really improved the study. All other suggestions have also been adressed in a satisfactory manner

(Remarks on code availability)

Reviewer #2

(Remarks to the Author)
Dear Authors,

Many thanks for providing a revised manuscript and such a detailed and easy-to-follow rebuttal. I feel as though all of my comments have been addressed and I wish you the best of luck with your next steps in publication.

(Remarks on code availability)
Yes the repo seems to be complete and it provides a README

Open Access This Peer Review File is licensed under a Creative Commons Attribution 4.0 International License, which permits use, sharing, adaptation, distribution and reproduction in any medium or format, as long as you give appropriate credit to the original author(s) and the source, provide a link to the Creative Commons license, and indicate if changes were made.
In cases where reviewers are anonymous, credit should be given to 'Anonymous Referee' and the source.
The images or other third party material in this Peer Review File are included in the article's Creative Commons license, unless indicated otherwise in a credit line to the material. If material is not included in the article's Creative Commons license and your intended use is not permitted by statutory regulation or exceeds the permitted use, you will need to obtain permission directly from the copyright holder.
To view a copy of this license, visit https://creativecommons.org/licenses/by/4.0/

POINT-BY-POINT RESPONSE TO REVIEWER COMMENTS

Reviewer #1 (Remarks to the Author):

The authors present here a timely and interesting study, on the fine scale effects of urban land cover on mosquito and bird populations and the effects on WNV ecology. This provides much needed data on possible effects of climate adaptive measures on arbovirus ecology. What has been lacking are high quality studies that do not only focus on mosquitoes (although they are also relatively scarce, especially in the context of climate change adaptation) also incorporate changes in wild bird diversity and abundance and subsequent effects on arbovirus outbreak risks. The authors show clear differences in WNV presence between urban microenvironments in Berlin, in 2023. The manuscript is clearly written and provides high quality, in-depth analyses of virological, weather and ecological data. However, a major drawback of the study is the fact that the study only describes data collected in 2023 and a relative small number of study sites. Incidence of WNV in 2024 in Berlin was also high. The study would significantly improve if 2024 data could also be added and/or if the correlations with bird biodiversity could be substantiated.

> ***Re:*** *We thank the reviewer for the appreciative and constructive feedback to our study. We have now included data from the same study sites from the mosquito season in 2024. Despite lower WNV infection rates, the observed differences in WNV abundance across urban microenvironments was consistent and confirmed our previous conclusions. With respect to bird abundance, we further conducted a field bird survey in spring 2025, repeating surveys four times per plot in accordance with the survey and data underlying the modelled abundance, and compared the model results using zero-inflated linear models (see Supplementary Figures 2-4, and Supplementary Methods). Interestingly, the model was well represented by the survey (see below; now Figure 6b); the sites RA and C – which had the highest WNV infection rates – also had the highest bird abundances, driven by the most common species such as great tits (Parus major) and wood pigeons (Columba palumbus) (Supplementary Figure 2). This strengthens our hypothesis on most common and abundant bird species to be involved in the cycling of the virus.*

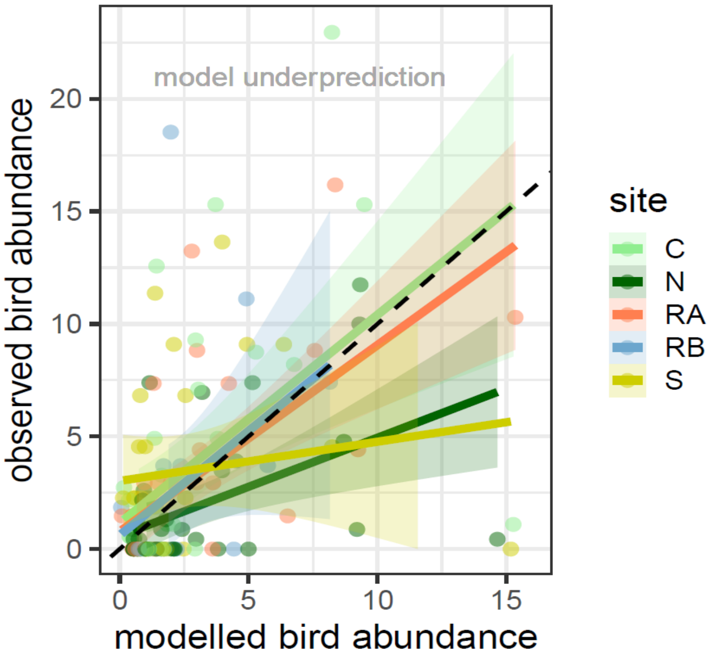

Some more detailed suggestions and questions:
Line 88: Here would be good to mention human responses to drought that can also increase vector abundance. Examples are irrigation practices and water storage (eg Hydrologic Conditions Describe West Nile Virus Risk in Colorado).

> ***Re:*** *Thank you very much for mentioning this. We agree with the reviewer that irrigation is an additional factor that may have a strong impact on WNV transmission. We added potential effects of anthropogenic responses to drought on WNV transmission and added the citation of the suggested study. The text now reads: "…whereas drought can increase contact between birds and mosquitoes at remaining natural water bodies as well as at anthropogenic water sources, such as irrigation and water storage sites."*

Line 165-168: "Mosquito MIR was positively correlated with open water and the abundance of the great tit (*Parus major*) and the common wood pigeon (*Columba palumbus*), as inferred from bloodmeal DNA". Some of this has also remained unclear in other parts of the manuscript: 1) did the predicted bird abundance correlate with results of blood meal analyses? 2) your sample size of bloodmeals is low – how reliable is this correlation?

> ***Re:*** *Indeed, the sample size of the bloodmeal analysis dataset is small. However, we could nearly double the vertebrate host sequences by including the 2024 dataset. A highly similar mosquito feeding pattern for the five microhabitats was found.*
> *With respect to 1) we can now happily show that an independent bird survey distinguished the sites exactly in high abundances of those species we also found in the bloodmeals (see answer above; general remarks reviewer #1 and Supplementary Information).*
> *With respect to 2), we included the low sample size of blood-fed mosquitoes in a limitation section in the discussion. The text now reads: "This study has some constraints that should be considered when interpreting the findings. Sampling was restricted to a single area comprising five microhabitats, and it remains to be determined whether similar patterns occur in other parts of the Berlin metropolitan area that include comparable microhabitat structures. Although consistent trends were observed across two consecutive sampling seasons, suggesting microhabitat-specific effects, confirmation in additional urban regions is required to assess generalizability. In addition, the number of blood-fed mosquitoes available for analysis was limited. More comprehensive inference regarding host–vector interactions would benefit from larger numbers of blood-fed specimens collected throughout the mosquito season and across microhabitats. Future studies integrating blood meal analyses with contemporaneous bird community assessments and systematic testing of birds for WNV infection at the same sites would clarify which avian species that contribute most to local WNV amplification and help disentangle microhabitat-specific transmission dynamics."*

Line 203-204: Previous literature seems to suggest much more intermixing of strains and less focal presence of WNV. Can you address that in this paragraph?

> ***Re:*** *We thank the reviewer for this comment. Systematic mosquito-based WNV surveillance studies that combine fine-scale spatial sampling across one season or multiple seasons with genomic analyses are still rare in Europe. Consequently, there is little empirical evidence regarding the extent of focal versus intermixed circulation at small spatial scales.*
> *In a previous study conducted in a rural area in eastern Germany, we observed higher rates of virus exchange between sites and less pronounced local amplification, although endemic maintenance without detected introductions from outside Germany was also evident. In contrast, the strong local amplification observed in the present urban study may reflect differences in host movement ecology. Urban bird populations are typically more residential and exhibit shorter movement distances than rural bird populations, potentially limiting virus dispersal and promoting microhabitat-level amplification.*

To clarify this, we have added the following text passage: "The extensive local amplification and maintenance of closely related variants over consecutive years support the importance of microhabitat-level ecological conditions and suggest more limited virus dispersal via infected birds in urban settings. In contrast, higher rates of dispersal of related virus variants have been observed in mosquitoes from rural areas of eastern Germany[66]. Notably, five WNV strains clustered within the broader genetic diversity of detected WNV sequences in Germany and were most likely introduced from other regions in the country. These sequences exhibited between 30-94 SNVs and were substantially more diverse than the 62 locally amplified strains, which showed 0 – 31 SNVs. We found no evidence of subsequent local amplification of these introduced strains. Variation in WNV infection rates between sites is therefore more likely driven by urban environmental characteristics rather than by virus introductions."

Line 231-232: What is not completely clear to me is whether you indeed also see higher bird diversity in the included green areas in this specific study?

Re: *Bird diversity data for the study sites were* modelled based on environmental descriptor variables and breeding bird censuses that *were conducted in Berlin but did not take place at the study sites. We thus do not have data on the actual bird diversity at the study sites. However, we now added data from a recent preliminary bird monitoring conducted at the study sites (see also answer and graph above; Supplementary Information). We could indeed confirm very good model fit for the sites C, RA, and RB. The two sites S and N had lower than expected bird abundances. Especially for the nature park area this is an interesting finding and maybe the explanation why we find lower infection rates here than for example in C and RA, which both contain large open standing water sources and high human density interacting (feeding) with the bird community.*

Line 240: *Culex* bioforms – include current literature showing that the distinction between biotypes is not so clear.

Re: *We added literature indicating that the distinction is controversial and included the following text passage: "This species occurs in two bioforms[75], with biotype pipiens feeding preferentially on birds and biotype molestus on humans, with hybrids discussed to acting as bridge vectors for bird-to-human transmission[76], even though recent studies have also contradicted this hypothesis[77,78]."*

Line 249: effectivity of vector control?

Re: *We agree with the reviewer that vector control is not feasible in the larger urban context. However, the focus here is on defined urban hotspot areas. Vector control at such sites, e.g. gardens and cemeteries, can effectively be implemented, e.g. by reducing larval breeding sites such as emptying catch basins. We changed "vector control" to "control of Culex larvae by reducing breeding sites" to emphasize non-pesticide control methods.*

Line 360-362: you base your selection of positive species on 3 papers, while the body of evidence of WNV exposure and infections in Europe is much larger. Did you apply a cutoff, or include all species positive at least once? IN literature there is evidence of at least 200 European species that have tested WNV positive – that may have impacted your analyses and results?

Re: *Thank you for this comment. We apologize if this was not clearly explained. We have conducted a comprehensive literature review and all bird species where WNV RNA was detected or anti-WNV reactive antibodies were detected, were considered susceptible (positive), even if species were only found positive once. As reference we have included three of the largest studies conducted in Europe on WNV in birds. Data for all bird species that*

occur in Berlin exist in the three studies. No cutoff was applied. However, for our analyses to identify variables that are associated with mosquito MIR, not all potentially WNV susceptible European bird species were included. The analyses were based on the following:
a. Correlation analysis (Figure 6): as shown in Supplementary Table 2, we used variables from a spatial model on local bird abundance that was based on a breeding bird census, environmental variables, such as temperature, tree cover, abundance and richness of mosquitoes and birds etc. We further included the information obtained from the mosquito bloodmeal analysis shown in Figure 4e. In brief, we included for all bird species that were considered WNV susceptible based on the literature review, how often mosquitoes were found to take a bloodmeal at this species per location (bloodmeal frequency).
b. Principal component analysis (Figure 7): We also conducted dimension reduction techniques (PCA) to analyze the relation between MIR (infection rate), Culex abundance, climatic and environmental predictor variables and 44 modelled bird species abundances. Here, the most prominent variable pointing to MIR – apart from Culex abundance as host species – was the presence of standing open water and bird species such as great tits (*Parus major*) and wood pigeons (*Columba palumbus*) *that were found to be the driving bird species behind differences in observed versus modelled bird abundances.*

The texts of the according methods and results sections have been revised to better explain the conducted analyses and we added the field survey confirming the bird model results. We have also added a limitations section to the discussion to indicate that our results have to be interpreted with caution and that future studies integrating blood meal analyses from a larger number of blood-fed mosquitoes combined with contemporaneous bird community assessments and systematic testing of birds for WNV infection at the same sites would clarify which avian species contribute most to local WNV amplification and help disentangle microhabitat-specific transmission dynamics.

I am missing a legend for figure 6

***Re:** A revised legend for Figure 6 is now included.*

Reviewer #2 (Remarks to the Author):

Thank you for the opportunity to review your manuscript titled: “Urban land cover drives fine-scale heterogeneity and extensive local amplification of West Nile virus”. Overall, it is a great paper and I want to congratulate the authors on excellent research. I think in the manuscript there are a few limitations missing, two that jumped out to me were the fact that you only looked at one summer and the potential sampling bias of your sites (would the results have changed a lot if you changed sites).

***Re:** We thank the reviewer very much for the time spent to review our manuscript and for the positive evaluation of our study. These limitations were mentioned by the Editor and other reviewer as well. To address this, we have now included data from a second summer, the mosquito season in 2024. In addition, a limitation section has been added to the discussion section that addresses the small number of sampling sites and the potential sampling bias of the five sites of our study.*

Below are some minor corrections.

Abstract:

Line 19: In some areas climate change and extreme events are actually decreasing mosquito-borne diseases, so perhaps it is more accurate to say, "In some areas climate change is…."

> ***Re:*** *We agree that this distinction is important. We have re-phrased the sentence and it now reads: "Climate change can intensify …").*

Introduction:
Line 88: Might be good just to give a very brief explanation of what you mean by a "sponge city"

> ***Re:*** *We have now added a short explanation to the concept: "Berlin provides a valuable case study, as it is actively implementing climate adaption measures, such as sponge city projects, an urban planning approach that enhances rainwater retention and infiltration through decentralised rainwater management, including the installation of permeable surfaces and natural drainage systems (Berliner Regenwasseragentur, https://regenwasseragentur.berlin/), the Green Roof program (Berliner GründachPLUS Programm, 2026-01), and the Berlin Tree Planting Law, which aims to plant over one million trees by 2040 (Berliner Klimaanpassungsgesetz, 2025-11-07)."*

Line 89: I think Nat Comms uses British, rather than American English, so it should be "decentralised"

> ***Re:*** *Thanks, this has been corrected.*

I then jumped to your methods first.

Methods:
Line 256: "which is with 892 km" is the "with" a typo? So the sentence would be" The study area is located in Berlin, which is 892 km2 and has a population of 3.8 million, the largest and most populous metropolitan area in Germany." The "has" was also missing.

> ***Re:*** *Thank you. We have now corrected the sentence: "The study area is located in Berlin, the largest and most populous metropolitan area in Germany, with an area of 892 km² and a population of 3.8 million."*

Line 272: Are you missing a "the" before "CO2-source"?

> ***Re:*** *This has been added.*

Line 293: Switch out "analyzed" for "analysed"

> ***Re:*** *This has been corrected.*

Line 342: Remove "in the", in the sentence, "variable values in the falling within…"

> ***Re:*** *This has been deleted.*

Data availability:
Line 393: Was the Berlin geoportal data also publicly available, and is there a code repo?

> ***Re:*** *The Berlin geoportal data is publicly available at the mentioned homepage. The code repository with all data, code and results is also publicly available as developer repository at https://github.com/EcoDynIZW/wnv_ecological-analyses_code-for-review and will be published together with the manuscript upon acceptance.*

Back to the results.

Results:
Line 99: Swap out "standardized" for "standardised"

> ***Re:*** *This has been corrected.*

Line 106: You say that WNV in the mosquitoes was "unexpectedly high", but do we have a good idea of what the infection rate is in Berlin mosquitoes? Why is this unexpected and did you have a baseline that you were comparing this to?

> ***Re:*** *We agree that this information is missing. We have now added references supporting this statement and revised the sentences: "In 2023, WNV infection in mosquitoes was unexpectedly high, with 4.8 % of mosquito pools testing positive (78/1,625). For all sites except N, minimum infection rates (MIRs) peaked in August, with the highest MIRs observed at C (20.77) and RA (14.60) (Table 2, Figure 2D, E), reaching levels comparable to those reported from outbreak areas in southern Europe[59-61]."*

Discussion:
Line 185: I would also add "Aedes albopictus" too.

> ***Re:*** *We agree and have now included Aedes albopictus.*

Line 187: Switch out "urbanization" with "urbanisation"

> ***Re:*** *This has been corrected.*

Line 193: I think here you need to back up this claim of "unexpected" - you need to link this to literature to understand why this would be unexpected, if the mosquito and host are abundant and you get frequent cases of WNV.

> ***Re:*** *We deleted "unexpected" here, as this is explained later in the discussion in lines 327 - 338. The text in the respective section reads: "Observed MIRs of this study were unexpectedly high in 2023 and comparable to European regions with recurring outbreaks and high numbers of human cases, e.g. Northern Italy and Central Greece[59-61]. This contrasts with the reporting of a single human WNV case in Berlin in 2023 and seven in 2024, suggesting underdiagnosis due to no or mild symptoms upon infection and low public and physician awareness, as well as the predominantly avian feeding behaviour of Cx pipiens. This species occurs in two bioforms[75], with biotype pipiens feeding preferentially on birds and biotype molestus on humans, with hybrids discussed to act as bridge vectors for bird-to-human transmission[76], even though recent studies have also contradicted this hypothesis[77,78]. Our blood-meal analyses provide evidence for occasional human exposure, and two autochthonous WNND cases were reported in proximity to our study sites in 2021 and 2022[55]. The high density and infection rates of WNV vectors at RA, with a substantial elderly population as highest risk group for severe disease, highlight the public health relevance of these findings[23]."*

Line 239: I would say underdiagnosis is more complex than that, probably with the most likely reason being mild infection and common febrile symptoms.

> ***Re:*** *Thank you for mentioning this. We have included this information in the text.*

Line 245: Perhaps it would be useful to state why the high elderly population would matter?

Re: *Thank you. This information has been added (see above).*
